# Apple Russet Ring and Apple Green Crinkle Diseases: Fulfillment of Koch’s Postulates by Virome Analysis, Amplification of Full-Length cDNA of Viral Genomes, *in vitro* Transcription of Infectious Viral RNAs, and Reproduction of Symptoms on Fruits of Apple Trees Inoculated With Viral RNAs

**DOI:** 10.3389/fmicb.2020.01627

**Published:** 2020-07-10

**Authors:** Chunjiang Li, Hajime Yaegashi, Ryusuke Kishigami, Ayaka Kawakubo, Noriko Yamagishi, Tsutae Ito, Nobuyuki Yoshikawa

**Affiliations:** ^1^Faculty of Agriculture, Iwate University, Morioka, Japan; ^2^Division of Apple Research, Institute of Fruit Tree and Tea Science, National Agriculture and Food Research Organization, Morioka, Japan; ^3^Agri-Innovation Center, Iwate University, Morioka, Japan

**Keywords:** apple russet ring disease, apple green crinkle disease, next generation sequencing, apple chlorotic leaf spot virus, apple stem pitting virus, Koch’ postulates

## Abstract

Apple russet ring and apple green crinkle are graft-transmitted diseases first reported more than 60 years ago, but at present, no association between a specific virus (variant) and the disease has been clearly demonstrated. In this study, we conducted the following series of experiments to identify the causal viruses (variants) of these apple diseases; (1) comprehensive analysis by next-generation sequencing of all viruses in each apple tree affected with russet ring or green crinkle disease, (2) amplification of full-length genomic cDNA of viruses using primers containing the T3 promoter and the *in vitro* transcription of infectious viral RNAs, (3) inoculation of viral RNA transcripts to both herbaceous and apple plants, (4) analysis of sequence variants of viruses present in infected plants, (5) back-inoculation of sequence variants of candidate viruses to apple seedlings combined with the virus-induced flowering technology using the apple latent spherical virus vector to reproduce the symptom on the fruit as soon as possible, and (6) reproduction of symptoms on the fruits of apple trees inoculated with sequence variants and the re-isolation of each virus variant from apples showing fruit symptoms. The results showed that one of the sequence variants of the apple chlorotic leaf spot virus causes a characteristic ring-shaped rust on the fruits of infected apple trees and that a sequence variant of the apple stem pitting virus probably causes green crinkle symptoms on an infected apple fruit. Thus, we were able to fulfill Koch’s postulates to prove the viral etiology of both the apple russet ring and green crinkle diseases. We also propose an experimental system that can prove whether a virus found in diseased tissues is the pathogen responsible for the diseases when the etiology is undetermined.

## Introduction

There are many virus-like diseases affecting deciduous fruit trees whose causal agents are not yet identified (Németh, 1986; Howell et al., 2011). Even if a certain virus is presumed to be the cause of the disease, the causal relationship between the presence of a virus and specific symptoms has not been conclusively proven in many fruit tree viral diseases because back-inoculation of the virus to the original fruit trees has not been achieved; that is, Koch’s postulate has not yet been fulfilled. There are several reasons why it is difficult to identify the causative virus of fruit tree diseases. Firstly, many viruses are not so easy to isolate into herbaceous test plants and/or to back-inoculate the isolated virus into their original fruit trees. Secondly, some viruses of fruit trees have not only difficulties to be transmitted to herbaceous host plant, but they simply have a limited host range. This makes it difficult to isolate the causative agent as a single virus. The third reason is that the infection pattern of viruses is often very complex in fruit trees. For example, reports show that a single cherry tree affected by a bud blight disease was infected with 7 – 8 different virus species at the same time, and that most of these viruses could not be transmitted and isolated in herbaceous test plants (Isogai et al., 2004; Yaegashi et al., 2020). In Japan, most apple trees are infected with viruses such as apple chlorotic leaf spot virus (ACLSV), apple stem pitting virus (ASPV), and apple stem grooving virus (ASGV), etc. without showing obvious symptoms (Yaegashi et al., 2011). In addition, each virus (ACLSV, ASGV, or ASPV) in single apple trees consists of a mixture of sequence variants that differ considerably from one another in terms of nucleotide sequences (Candresse et al., 1995; Yoshikawa et al., 1996, 2001; Magome et al., 1997b, 1999; Yaegashi et al., 2007). The complexity of the virus infection patterns in fruit trees makes it difficult to identify the virus or virus variant responsible for a specific disease. Examples of graft-transmitted diseases of apples with undetermined etiology include the apple russet ring and apple green crinkle diseases (Howell et al., 2011).

The apple russet ring is a graft-transmitted disease of apple in which a characteristic ring-shaped rust appears on the fruits. The disease was firstly reported on a ‘McIntosh’ apple that showed leaf pucker symptoms in 1954 in British Columbia, Canada (Wood, 1971). The russet ring symptom of the fruit was shown to be associated with leaf pucker (Wood, 1971). The graft transmission of the russet ring disease was reported on the ‘Golden Delicious’ apple from Washington state, United States (Wood, 1971); in Japan, the russet ring disease was found in the ‘Mutsu’ apple from Iwate Prefecture in 1972 and was also shown to be grafted-transmitted (Yanase et al., 1988). The disease is now reported to occur worldwide and implicated to be caused by a strain (or by strains) of ACLSV (van der Meer, 1986; Desvignes and Boye, 1988; Wood, 2001; Howell et al., 2011). However, many strains or isolates of ACLSV are naturally in apple trees that are both unaffected and affected by russet ring disease (Yaegashi et al., 2007, 2011). In order to demonstrate that ACLSV may be responsible for apple russet ring disease, it is necessary to isolate and characterize the strain and its sequence variants at the molecular level, and to back-inoculate these into apple plants for symptom reproduction.

Apple green crinkle disease was first observed in 1929 in Aomori Prefecture, Japan and the graft-transmission of this disease was demonstrated in Japan in 1934 (Sawamura, 1965), thus this disease is the world’s first reported virus-like disease affecting apple plants. The disease was also reported in Canada and New Zealand in the 1930s (Chamberlain et al., 1974). Symptoms occur only in the fruit and appear most clearly during the young fruit stage, wherein some of the fruit are not enlarged and are irregularly dented, resulting in abnormal fruit morphology. The pericarp of the indented part will later cork and become rust-like, and in severe cases, it will tear (Chamberlain et al., 1974). To date, multiple viruses such as ASPV, ACLSV, and ASGV etc. have been detected in trees affected by green crinkle disease (Yaegashi et al., 2007; James et al., 2013). Among them, ASPV has been implicated as the causal virus, but the relationship between ASPV isolates and crinkle symptoms of fruits has not been demonstrated (Howell et al., 2011). James et al. (2013) reported that a putative new foveavirus closely related to ASPV, namely the apple green crinkle associated virus (AGCaV), had been isolated from apples showing severe crinkle disease.

In this study, we conducted the following series of experiments to determine the causal viruses (variants) of the apple russet ring and green crinkle diseases: (1) comprehensive analysis by next-generation sequencing (NGS) of all viruses in each apple tree affected by russet ring or green crinkle diseases, (2) amplification of the full-length genomic DNA of viruses using primers containing the T3 promoter and the *in vitro* transcription of infectious viral RNAs, (3) inoculation of virus RNA transcripts to herbaceous and apple plants, (4) analysis of sequence variants of viruses in infected plants, (5) back-inoculation of sequence variants of candidate viruses to apple seedlings combined with virus-induced flowering technology using the apple latent spherical virus (ALSV) vector (Yamagishi et al., 2014, 2016; Yamagishi and Yoshikawa, 2016), and (6) reproduction of symptoms on the fruits of apple trees inoculated with sequence variants and the re-isolation of each virus variant from apples showing fruit symptoms. From the results of the above studies, we were able to prove the specific sequence variants of the causal viruses of the two apple diseases. Our study establishes an experimental system to identify the causal virus of fruit tree diseases with undetermined etiology by isolation of the viruses using the amplification of full-length cDNA of viral genomes followed by *in vitro* transcription of infectious viral RNAs and reproduction of symptoms on original hosts by back inoculation.

## Materials and Methods

### Plants and dsRNA Extraction

An apple tree (PK-51, ‘Royal Gala’) affected with the apple russet ring disease (ARRD) and an apple tree (P-190, ‘Golden Delicious’) with the apple green crinkle disease (AGCD) were maintained in the field of the Division of Apple Research, Institute of Fruit Tree and Tea Science, NARO in Morioka, Iwate prefecture, Japan. These apple trees show typical symptoms of russet-ring and green crinkle on fruits, respectively (Supplementary Figure S1). dsRNAs were extracted from the newly developed leaves of an apple tree PK-51 infected with ARRD and an apple tree P-190 infected with AGCD. Leaf samples (20 g) were collected from diseased apple trees and stored at −80°C until use. Extraction of dsRNA from the leaves of diseased trees were performed using cellulose powder as described previously (Morris and Dodds, 1979; Yoshikawa and Converse, 1990), and the dsRNA was dissolved in 20 μL TAE buffer (40 mM Tris, 20 mM sodium acetate, 1 mM EDTA, pH 7.0). The dsRNA was electrophoresed on a 5% polyacrylamide gel and stained with silver nitrate to determine the presence of dsRNAs (Yoshikawa and Converse, 1990).

### Next-Generation Sequencing and Computational Analysis

dsRNAs from the ARRD or AGCD-infected samples were then subjected to library preparation as reported previously (Noda et al., 2017; Yaegashi et al., 2020). The prepared library was analyzed using 6% polyacrylamide gel electrophoresis and the products (200–300 bp) were purified and subjected to paired-end sequencing (read length 101 bases) using the HiSeq2000 (Illumina Inc., San Diego, CA, United States). Resulting reads were assembled *de novo* into contiguous sequences (contigs) using Velvet (ver. 1.2.08) (Zerbino and Birney, 2008) with *k* = 97 in RR and *k* = 95 in GC samples. The obtained contigs (>500 bp) were then used to search for virus sequences using the blast program of NCBI BLAST+.^1^

### RT-PCR

Leaves and petals were collected from apple trees affected by ARRD (PK-51) and AGCD (P-190) and were stored frozen at −80°C. Frozen samples (1 g) of each affected tree were put into a 2.0 mL microtube together with stainless steel beads SUB-50 (TOMY SEIKO Co., Ltd., Tokyo, Japan) and added 800 μL of RNA extraction buffer [2% CTAB, 2% PVP K-30, 100 mM Tris-HCl (pH 8.0), 25 mM EDTA (pH 8.0), 2 M NaCl, 2% mercaptoethanol]. Samples in microtubes were crushed using a Micro Smash MS-100R (TOMY SEIKO) and incubated at 65°C for 20 min with occasional stirring. After the addition of 800 μL of chloroform, the extracts were sufficiently stirred and centrifuged at 10,000 × *g* for 10 min (4°C). Then, 700 μL of the supernatants were added to 233 μL of 7.5 M LiCl, sufficiently mixed, and left at 4°C overnight. After centrifugation at 14,000 × *g* for 25 min, the pellets were added to 1 mL of 80% ethanol and centrifuged at 14,000 × *g* for 5 min. The pellets were then suspended in sterilized distilled water (SDW) to obtain the total RNA solutions. Total RNA concentrations were measured by NanoDrop (Thermo Fisher Scientific, Tokyo, Japan).

The coat protein (CP) region of each virus was amplified through RT-PCR as follows: 1 μL of total RNA solution (1 μg/μL), 0.5 μL of Oligo (dT)*12* primer (10 μM), 4 μL of 2.5 mM dNTP mixture (TaKaRa Bio Inc., Kusatsu, Japan), 2 μL of 5 × RT Buffer (TOYOBO Co., Ltd., Tokyo, Japan), 0.5 μL of ReverTra Ace (100 unit/μl) (TOYOBO), and 2 μL of SDW were mixed and incubated at 42°C for 60 min using the TaKaRa PCR Thermal Cycler Dice^®^ Version II (TaKaRa) for reverse transcription reaction. PCR was then conducted using TaKaRa Ex Taq and the following primer pairs: SP-CP8148-8167 (+) [5′-ttcgaccctaaccttcatgg-3′] and SP-CP8870-8890 (–) [5′-ctttgagtttgcagcatgagg-3′], with the expected product corresponding to base numbers 8148 to 8890 of ASPV-IF38 (accession no. AB045371) (Yoshikawa et al., 2001) for the ASPV-CP region, AC-CP 6821-6840 (+) [5′-agatctgaaagcgttcctg-3′] and AC-CP7342-7365 (–), with the expected product is corresponding to bases 6821–7365 of ACLSV-P205 (accession no. D14996) (Sato et al., 1993) for the ACLSV-CP region, and SG-CP5590-5611(+) (5′-aaagagaaagtttaggtccctc-3′) and SG-CP6395-6413 (–) (5′-taaaggcaggcatgtcaac-3′) corresponding to bases 5590–6413 of ASGV-P209 (accession no. D14995) (Yoshikawa et al., 1992). The PCR conditions were: 35 cycles of 94°C for 30 s, 58°C for ASPV, 54°C for ACLSV, and 55°C for ASGV for 30 s, 72°C for 1 min. PCR products were electrophoresed on a 1% agarose gel, purified using MonoFas^®^ DNA purification kit (GL sciences), and then TA cloning was performed using the TaKaRa Mighty TA-cloning Kit (TaKaRa). Plasmids were extracted from *Escherichia coli* and used for sequence analysis as descried below.

### *In vitro* Transcription of Virus Genomic RNA

Synthesis of full-length DNA from the genomic RNAs of ACLSV, ASPV, and ASGV was done by subjecting total RNA samples to a reverse transcription reaction at 42°C for 60 min using the PrimeScript II RT enzyme and oligo dT primer from the PrimeScript^®^ High Fidelity RT-PCR Kit (TaKaRa) according to manufacturer protocols. Full-length DNAs of the three viruses were amplified by PrimeSTAR GXL DNA polymerase (TaKaRa) using primer pairs containing the T3 promoter sequence: ACLSV5’endT3(+) (5′-aattaaccctcactaaagtgatactgatacagtgtacact-3′) and ACLSV3’end(–) (5′-tttttttagtagtaaaatatttaaaagt-3′) for ACLSV, ASPV5′endT3(+) (5′-aattaaccctcactaaaggatacgcaaacaaactctg-3′ and ASPV3′end(−) (5′-ttttttgaaaatctagttaaaacaaaaataag-3′) for ASPV, and ASGV5′ endT3(+) (5′-aattaaccctcactaaaggaaatttaacaggcttaattt-3′) and ASGV3′end(−) (5′-ttttttttttttttttttttagagtggacaaactctagac-3′) for ASGV. PCR was performed as follows: 98°C for 30 s, 54°C for ACLSV, 59°C for ASPV, and 52°C for ASGV for 30 s, and 68°C for 10 min for 40 cycles. The PCR product was then purified using the MonoFas^®^ DNA Purification Kit II (GL Sciences Inc., Tokyo, Japan). Purified full-length genomic DNAs were then used as templates for the *in vitro* transcription of full-length genomic RNA of each virus using the MEGAscript^®^ Kit (Ambion, Thermo Fisher scientific, Tokyo, Japan). The reaction mixtures contained the full-length genomic cDNA of each virus, ATP, CTP, UTP (75 mM each), GTP (15 mM), Cap Analog (40 mM) Enzyme Mix, and RNasin^®^ Plus RNase Inhibitor (Promega, Madison, United States), and were incubated for 3.5 h at 37°C. The reaction mixtures were then treated with TURBO DNase (Thermo Fisher scientific) (2 U/μl) at 37°C for 15 min to decompose the template DNAs. RNA samples were then extracted using water-saturated phenol and chloroform, precipitated using ethanol, and suspended in SDW. RNA transcript concentrations were measured using NanoDrop, while RNA transcript quality was also checked using 1% agarose gel electrophoresis.

### Inoculation of RNA Transcripts to Plants and Sequence Analysis of Virus Variants in Infected Plants

Inoculation of the RNA transcripts of ACLSV, ASPV, and ASGV to *Nicotiana occidentalis* was conducted as follows: two leaves of *N. occidentalis* plants (6th to 7th leaf stage) were inoculated with 10 μL of viral RNA transcripts (0.5–1 μg/μl) per leaf, along with carborundum. After inoculation, plants were grown in a growth chamber at 25°C with the day length set to 8 h. *Chenopodium quinoa* was also used for inoculation of the ACLSV-RNA transcript.

Total RNA was extracted from the leaves of infected plants and the CP region of each virus was amplified as described above. For sequence analysis of virus variants in each infected plant, the PCR products were purified using MonoFas^®^ DNA purification kit (GL sciences) and TA cloning was performed using the TaKaRa Mighty TA-cloning Kit (TaKaRa). DNA plasmids were purified using a Plasmid DNA Extraction Mini Kit (FAVORGEN Biotech, Ping-Tung, Taiwan) according to the manufacturer’s protocol. The sequences of the cDNA clones were analyzed using a DNA sequencing service from Sigma-Aldrich Japan. The homology of the CP region sequence between DNA clones was analyzed using the DNASIS software (Hitachi, Tokyo, Japan).

### Back-Inoculation of Apple Seedlings With ACLSV and ASPV

Both ARRD and AGCD cause characteristic symptoms on apple fruits as shown in Supplementary Figure S1. However, it generally takes at least 6–7 years for apple seedlings to bloom. For this reason, we used virus-induced flowering (VIF) system using the ALSV vector (Yamagishi et al., 2014, 2016; Yamagishi and Yoshikawa, 2016) for symptom reproduction on the apple fruits. VIF using the ALSV vector can reduce flowering 2 months after germination and lead to fruit formation within 1 year in apple seedlings (Yamagishi et al., 2014).

Total RNA samples extracted from infected *N. occidentalis* leaves were used as templates for generating full-length cDNA of each sequence variant of both ACLSV and ASPV, followed by *in vitro* transcription of infectious viral RNA, as described above. As inoculum sources for inoculation to apple seedlings, we selected five *N. occidentalis* plants infected with RRACV1, RRACV2, RRACV3, RRACV5, or RRACV1 + RRACV4. In the case of ASPV variants, the RNA transcripts of ASPV from the AGCD-sample were also inoculated to apple seedlings with the ALSV-AtFT/MdTFL1 vector through particle bombardment, as described above.

Immediately after germination, cotyledons of apple seedlings from seeds of ‘Ourin’ were biolistically inoculated with the RNA transcript of each viral RNA, along with the RNA of ALSV-AtFT/MdTFL1 as described previously (Yamagishi et al., 2010, 2016). Inoculation was performed in a helium pressure of 1379 kilopascal (kPa) using the Helios Gene Gun system (Bio-Rad Laboratories, München, Germany), with two shots used per cotyledon (4 shots in total). Inoculated apple seedlings were incubated in a growth chamber at 25°C for ACLSV and at 20°C for ASPV, with the day length set to 16 h for approximately 2 months, and then grown in a non-contaminant greenhouse.

Infection assay was conducted by RT-PCR as described before (Yamagishi et al., 2014) using primer pairs for ACLSV [ACCP6821-6840(+) (5′-agatctgaaagcattcctg-3′) and ACCP7342-7365(−) (5′-ctaaatgcaaagatcagttgtaac-3′)], ASPV [SPCP8148-8167(+) (5′-ttcgaccctaaccttcatgg-3′) and SPCP8 870-8890(−) (5′-ctttgagtttgcagcatgagg-3′)], ALSV-RNA1 [ALSV RNA16598(+) (5′-gtacattcctcccaatcaaag-3′) and ALSVRNA 16691(–) (5′-ggatcaggagaacaaactag-3′)], and ALSV-RNA2 [ALS VRNA21418(+) (5′-cccaaatctgctagaaggtc-3′) and ALSVR NA21511(–) (5′-gcaaggtggtcgtga-3′)]. When the infected apple seedlings flowered, they were pollinated to produce fruits.

### Grafting

To confirm that RRACV2 induced ARRD in apple seedlings as described above, graft-transmission tests were conducted using apple seedlings infected with each RRACV and ALSV-AtFT/MdTFL1. Approximately 1 year after inoculation by particle bombardment inoculation, the shoots of infected apple seedlings infected with each sequence variant (SV) of ACLSV were cut and grafted to the stem of 3-year-old virus-free apple trees (‘Golden Delicious’/JM7) using the side-grafting method for the reproduction of russet ring symptoms on the fruits. Grafted apple trees were named APT1 (an apple tree grafted with RRACV1), APT2 (with RRACV2), APT3 (with RRACV3), APT1 + 4 (with RRACV1 + RRACV4), and APT5 (with RRACV5). Inoculated apple plants were grown in a greenhouse for 4 years at the Apple Research Center, NARO in Morioka, Japan.

### Nucleotide Sequencing of the Complete Genome of ACLSV and ASPV Variants

DNA fragments covering entire genomes of the sequence variants of both ACLSV and ASPV were amplified from samples infected with each variant using the primer pairs shown in Supplementary Tables S1, S2. TA cloning of the amplified sequences was conducted as described above. Nucleotide sequencing of the cDNA clones was performed using a DNA sequencing service (Sigma-Aldrich, Japan).

## Results

### Virome Analysis of Apple Trees Affected by ARRD and AGCD

Using NGS analysis of dsRNAs (Supplementary Figure S2) from the ARRD and AGCD samples in this study, we obtained 51,514,584 reads from the ARRD sample and 70,424,624 reads from the AGCD sample (Table 1). The conventional *de novo* assembly of the reads from ARRD and AGCD samples generated 411 contigs with an average of 2,095 base pairs (bp) and 853 contigs with an average of 771 bp, respectively (Table 1). In a subsequent blastn analysis, 63 contigs from the ARRD-sample and 244 contigs from the ACGD-sample corresponded to virus sequences (Table 1). In the ARRD sample, ACLSV had the largest number of contigs (42.9% of the total), followed by ASPV (13%), ASGV (6.4%), AGCaV (3.2%), and apricot latent virus (ApLV) (1.6%) (Table 1). On the other hand, in the GC sample, ASPV was the largest, accounting for 34.4% of the total, followed by ASGV (8.2%), ACLSV (7.8%), AGCaV (2.9%), and ApLV (2.9%) (Table 1).

**TABLE 1:** Summary of the conventional *de novo* assembly strategy of the reads from russet ring (RR)- and green crinkle (GC)-diseased apple samples.

**Apple trees**	**No. of reads**	**Total no. of contigs**			No. of contigs with hit to virus**					
		**Total (mean, bp)**	With virus hit*	**With no hit**	**ACLSV**	**ASGV**	**ASPV**	**AGCaV**	**ApLV**	**others**
PK-51 with RR	51,514,584	441 (2,095)	63	378	27	8	11	2	1	14
P-190 with GC	70,424,624	853 (771)	244	609	19	20	84	7	7	107

*No. of contigs with hit to viruses with significant *E*-value (<0.001). **ACLSV, apple chlorotic leaf spot virus; ASGV, apple stem grooving virus; ASPV, apple stem pitting virus; AGCaV, apple green crinkle-associated virus; ApLV, apricot latent virus; others, stealth virus 1 etc.

### Isolation of Viruses Into Separate Plants by Inoculation of *in vitro* Transcripts of Viral RNAs

Virome analysis by NGS revealed that both PK-51 and P-190 trees were infected with multiple viruses as described above. In order to isolate each virus from a diseased apple trees into separate herbaceous plants, the full-length DNA of the genomic RNA of each virus was amplified from the total RNA of an apple sample with ARRD (PK-51) using RT-PCR (Supplementary Figure S3), and the obtained full-length viral RNAs were transcribed *in vitro* using the full-length DNAs as templates. It can be seen that RNAs corresponding to the size of each virus genome [ASPV, 9.3 kb: ACLSV, 7.4 kb; ASGV, 6.4 kb] were synthesized (Supplementary Figure S3). Each RNA transcript was inoculated into *N. occidentalis* plants, and the infection was assayed using RT-PCR, from which it was found that infection was present in 17 plants of the 24 plants inoculated with the ACLSV RNA transcript (infection rate, 71%), 5 out of 10 plants inoculated with ASPV (50%), and 6 out of 6 plants inoculated with ASGV (100%). Infected *N. occidentalis* plants showed symptoms consisting of mosaic and malformation of leaves (ACLSV) and of interveinal chlorosis (ASPV) (Supplementary Figure S4). *C. quinoa* plants infected with ACLSV showed chlorotic spots and mosaic in the upper leaves, which is typical of ACLSV infection (Supplementary Figure S4). *N. occidentalis* plants infected with ASGV did not show any obvious symptoms. RT-PCR using a primer pair for each virus showed that only ASPV was detected in plants inoculated with the ASPV RNA transcript, and the other two viruses (ACLSV and ASGV) were not detected (Supplementary Figure S3). Similarly, only ACLSV was detected in plants inoculated with the ACLSV RNA transcript (Supplementary Figure S3), and only ASGV was detected in plants inoculated with the ASGV-RNA transcript (Supplementary Figure S3). Thus, by the inoculation of *in vitro* transcripts of viral RNAs, each of the three viruses co-infecting an apple tree could be isolated into separate plants.

### Complexity of Sequence Variants of ACLSV in an Apple Tree (PK-51) Affected by ARRD

We analyzed the nucleotide sequence of the selected 18 clones of the ACLSV-CP region amplified from the ARRD-infected apple tree (PK-51). Here, those with nucleotide sequence identity of 99% or more in the CP region (582 nt) were regarded as deriving from the same SV. The results indicate that at least six sequence variants (SV1∼SV6) were present in samples from an apple with ARRD (Supplementary Figure S5). The detection frequency of each SV was different and the detected numbers of each SV in the total 18 clones analyzed were 5 clones for SV1, 2 clones for SV2, 1 clone for SV3, 4 clones for SV4, 6 clones for SV5, and 1 clone for SV6. Sequence identities of the CP region between SVs of ACLSV were 83.3–95.1% in terms of nucleotides and 91.7–97.9% in terms of amino acids.

**FIGURE 1:**
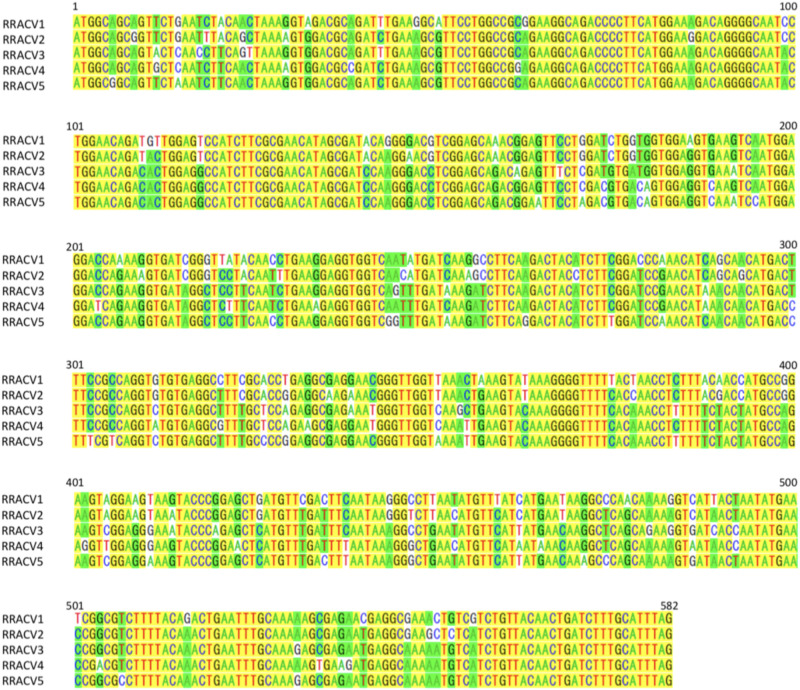
Alignment of the nucleotide sequences of CP region of ACLSV-RRACVs detected in leaf samples of *N. occidentalis* plants inoculated with the RNA transcript of ACLSV from ARRD. Identical bases among the five SVs are filled in yellow, and if there are substitutions in the bases, they are filled in green (the number is dominant) or unfilled (the number is inferior).

In subsequent experiments, we analyzed the SVs in 17 herbaceous plants (*N. occidentalis* and *C quinoa*) infected with the RNA transcript of the ACLSV genome. Nucleotide sequence analysis of DNA clones from 17 infected plants (10 clones/plant) indicated that five SVs (named RRACVs1 ∼ 5) were detected in infected plants (Figure 1). Among the 17 infected plants, most *N. occidentalis* plants were singly infected with either RRACV1 (11 plants), RRACV2 (2 plants), RRACV3 (1 plant), or RRACV5 (2 plants), except for a plant infected with RRACV4 that was found to be co-infected with RRACV1. Comparison of the RRACV1–RRACV5 nucleotide sequences with those of SV1-SV6 from an RR-apple in Supplementary Figure S5, RRACV1 was the same as SV1, RRACV2 was SV2, RRACV3 was SV3, and RRACV4 was SV4. On the other hand, RRACV5 was different from both SV5 and SV6 in an apple with ARRD, suggesting that at least seven SVs were co-infecting the RR-apple (PK-51). We could not find *N. occidentalis* plants infected with SV5 and/or SV6 in this experiment. Sequence identities of the CP region among RRACVs were 83.8–92.4% in terms of nucleotides and 91.7–97.9% in terms of amino acids (Table 2). The sequence identities between RRACVs 1-5, SVs 5 and 6, and isolates reported previously (P205 and B6) are shown in Table 2. It has been reported that ACLSV can be divided into two types (P205 and B6) based on the CP amino acid sequence (Yaegashi et al., 2007). Among the seven SVs found in an ARRD-apple and inoculated *N. occidentalis*, three SVs (RRACV3, RRACV4, and RRACV5) belong to the P205 type and four (RRACV1, RRACV2, SV5, and SV6) belong to the B6 type.

**TABLE 2:** Identities of the nucleotide and amino acid sequences of the coat protein gene between the sequence variants (SV) and the isolates of ACLSV found in infected *N. occidentalis* and an apple tree (PK-51) with ARRD.

**SVs and isolates**	**SVs and isolates**								
	**RRACV1**	**RRACV2**	**RRACV3**	**RRACV4**	**RRACV5**	**SV5**	**SV6**	**P205**	**B6**
RRACV1		91.0	85.4	83.8	85.0	95.1	93.4	84.5	91.2
RRACV2	97.9		87.1	86.0	85.5	91.7	89.1	85.2	96.0
RRACV3	93.2	92.7		91.7	92.4	86.4	85.7	89.7	86.6
RRACV4	92.2	91.7	97.4		91.7	84.8	83.3	87.1	86.4
RRACV5	97.9	91.7	97.9	92.2		84.7	82.8	88.4	86.2
SV5	97.9	96.8	92.7	91.7	91.7		92.7	83.7	90.5
SV6	92.2	97.4	91.7	97.9	90.6	96.3		83.6	88.3
P205	91.7	91.7	96.3	94.3	96.4	90.7	90.1		85.0
B6	97.9	97.9	94.3	92.7	92.7	97.4	96.4	90.2	

*The percentages of nucleotide sequence (582 bases) identities are above the diagonal and the percentages of amino acid sequence (193 aa) identities are below the diagonal. RRACV1∼5 was found in infected *N. occidentalis*, and both SV5 and SV6 was found in an ARRD-apple. P205 is an isolate from apple (Sato et al., 1993) and B6 is an isolate from an ARRD-apple (Yaegashi et al., 2007).*

### ACLSV-RRACV2 Induces Russet-Ring Symptoms on Fruits of Infected Apple Seedlings

Approximately 1 month after inoculation with viral RNA transcripts of RRACV1, RRACV2, RRACV3, RRACV5, and RRACV1 + 4 (10–20 seedlings per each RRACV), the upper leaves of apple seedlings were sampled, and virus infection was examined by RT-PCR. Both ACLSV and ALSV were detected in most of the inoculated apple seedlings (example shown in Supplementary Figure S6). However, some seedlings in which the *AtFT* gene was deleted from the ALSV vector were observed as shown in Supplementary Figure S6. As a result, 10 seedlings infected with RRACV1, 6 seedlings with RRACV2, 4 seedlings with RRACV3, and 4 seedlings with RRACV5 were used for further experiments. Infected seedlings showed early flowering from approximately 2–3 months after inoculation, and the flowered plants were artificially pollinated. Finally, the number of apple seedlings with fruits was found to be one with RRACV1, two with RRACV2, two with RRACV3s, and one with RRACV5. We could not obtain apple seedlings with fruits infected with RRACV1 + 4.

**FIGURE 2:**
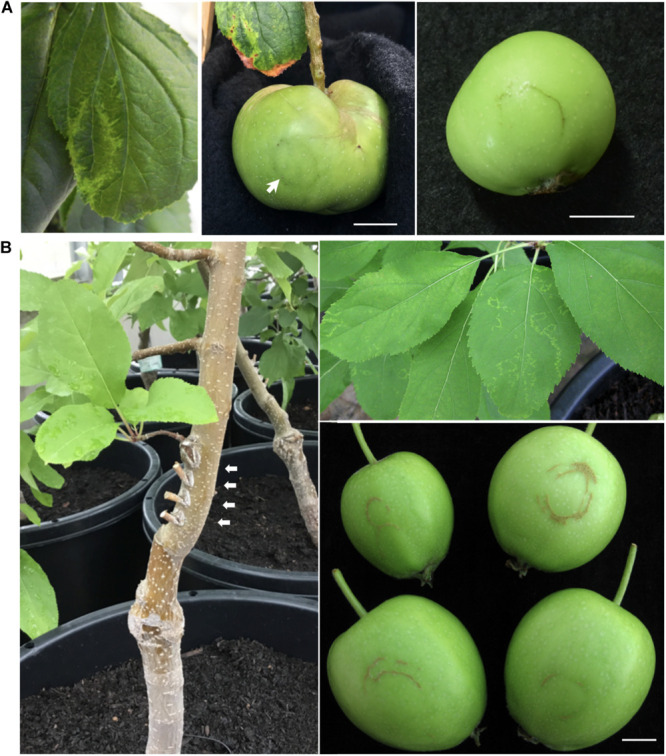
Symptoms of the apple plants inoculated with ACLSV-RRACV 2. **(A)** Ring pattern of chlorosis on a leaf (left) and ring pattern on the fruits (center and right) of apple seedlings co-inoculated with RRACV2 and ALSV-AtFT/MdTFL1. **(B)** An apple tree (‘Golden delicious’) 3 years after grafting with cut branches infected with ACLSV-SV. Arrows in a left photo indicated the positions of grafting. Ring and line patterns of chlorosis on leaves of an apple tree (AP2) infected with ACLSV-RRACV2 (upper right) and young fruits showing russet-ring symptom on an apple tree (AP2) infected with RRACV2 three years after grafting (lower right). Scale bar; ca. 1 cm.

Among the infected apple seedlings, a plant inoculated with RRACV2 (No. 1) showed a ring and line pattern of chlorosis on the leaf about 2 months after inoculation (Figure 2A, left). The fruits of the pollinated seedlings grew to 5–6 cm in diameter 5 months after pollination. In plant No. 1 infected with RRACV2, a dark green ring was observed on the fruits as shown in Figure 2A, center. Ring-shaped rust was observed on the fruit of another seedling infected with RRACV2 (no. 2) (Figure 2A, right). Though these apple seedlings were co-infected with ACLSV-RRACV2 and ALSV-AtFT/MdTFL1, ALSV-AtFT/MdTFL1 has never caused any symptoms in both leaves and fruits of infected apple seedlings (Yamagishi and Yoshikawa, 2016), and it is most likely that the symptoms observed were induced by RRACV2 infection. No symptoms were observed on either leaves or fruits of apple seedlings infected with RRACV1, RRACV3, or RRACV5. RT-PCR assays indicated that ACLSV was detected in fruit peel and flesh tissues of apple seedlings infected with RRACV1, RRACV2, RRACV3, or RRACV5, regardless of whether symptoms were observed.

### ACLSV-RRACV2 Causes ARRD in Apple ‘Golden Delicious’

All grafted apple trees (APT1 with RRACV1, APT2 with RRACV2, APT3 with RRACV3, APT1 + 4 with RRACV1 + RRACV4, and APT5 with RRACV5) produced fruits from a year after grafting, but no symptoms were observed on either leaves or fruits. Two years after grafting, the apple tree inoculated with RRACV2 (APT2) showed virus-like symptoms consisting of ring and line patterns of chlorosis on leaves as shown in Figure 2B; however, no symptoms were found on the fruits obtained this year. Three years after grafting, russet-ring symptoms typically of ARRD were observed on the fruits of APT2, in addition to ring- and line-patterns on leaves (Figure 2B). Other apple trees (APT1, APT3, APT1 + 4, APT5, and non-grafted control apple plants) did not show any symptoms on either leaves or fruits even 4 years after grafting. Thus, only apple trees inoculated with RRACV2 showed russet ring symptoms typical of ARRD. Amplification of the CP regions by RT-PCR and sequence analysis of the DNA products (6 clones per apple plant) from APT1, APT2, APT3, APT1 + 4, and APT5 indicated that only RRACV1 was detected from APT1, only RRACV2 from APT2, only RRACV3 from APT3, and only RRACV5 in APT5 (Supplementary Table S3). On the other hand, only RRACV1 was amplified from APT1 + 4, suggesting that RRACV4 might be lost from APT1 + 4 (Supplementary Table S3). ALSV-AtFT/MdTFL1 was not detected in any of the leaves of all apple trees (APT1–APT5) because we found that it is very difficult to transmit ALSV from infected to uninfected apple trees by grafting (unpublished result). From these results, we concluded that ACLSV-RRACV2 is a causal agent of ARRD.

**FIGURE 3:**
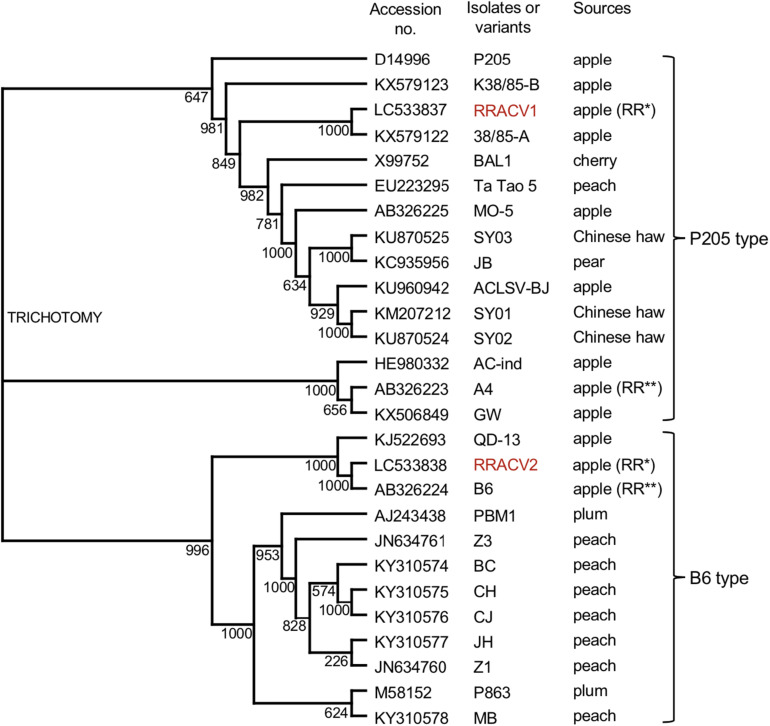
Phylogenetic tree based on the complete nucleotide sequences of ACLSV genomes. The tree was generated by CLUSTALW program (DNA databank of JAPAN [DDBJ]; URL: http://clustalw.ddbj.nig.ac.jp/). Bootstrap values for each node are shown (1000 replicates). Sequence variants analyzed in this study are shown in red. Apple (RR*) indicated an apple tree affected with ARRD used in this study and apple (RR**) is an another apple tree affected with ARRD described before (Yaegashi et al., 2007).

We have determined the complete nucleotide sequences of the RRACV2 and RRACV1 genomes. The genome of RRACV2 consists of 7557 nt excluding the 3′-polyA tail (GenBank/EMBL/DDBJ accession LC533838) and encodes three proteins from the 5′-terminus: an RNA replication-associated protein (Rep) [216 kilo dalton (kDa), 1883 aa], a movement protein (MP) (50 kDa, 460 aa), and a CP (21 kDa, 193 aa). The genome of RRACV1 consists of 7562 nt (accession LC533837) and contains three ORFs encoding a Rep (216 kDa, 1885 aa), an MP (50 kDa, 460 aa), and a CP (21 kDa, 193 aa). Phylogenetic analysis based on the complete nucleotide sequence of the ACLSV isolates showed that RRACV2 is grouped into the B6 type and RRACV1 into the P205 type (Figure 3). The RRACV2 genome sequence was closest to that of B4 isolated from another apple tree affected by ARRD, and the identities of nucleotide and amino acid sequences between RRACV2 and B6 were 89.4% (complete genome), 94.3% (Rep), 93.9% (MP), and 98.0% (CP).

### Eight ASPV-SVs Were Detected in an Apple Tree (P-190) Affected by AGCD

RT-PCR analysis of inoculated *N. occidentalis* plants showed that all plants (10 out of 10 inoculated plants) were infected with ASPV (Supplementary Figure S7). The PCR products (745 bp of the CP region of the ASPV genome) were purified from 10 infected plants and analyzed by direct sequencing using the SPCP8148-8167(+) and SPCP8870-8890(−) primer pair. We expected that some plants were infected with a single different SV, as was the case with RRACV; however, all *N. occidentalis* plants were found to be infected with multiple SVs, judging from the fluorescence peaks in the sequences of the amplified DNA (data not shown). Next, the amplified DNAs from 5 infected *N. occidentalis* plants were cloned as described above, and the resulting DNA clones were sequenced (7–10 DNA clones per infected plant). Among apple seedlings infected with both the ASPV-SV and ALSV-AtFT/MdTFL1 vector, we selected five seedlings for the amplification of their CP region, followed by cloning of the DNA products; DNA clones (13–18 clones per infected apple seedling) were also sequenced. Sequence analysis of DNA clones from 10 infected plants (5 *N. occidentalis* and 5 apple seedlings each) indicated that a total of eight SVs were detected in plants inoculated with RNA transcripts from apple (P-190) affected by AGCD. The SVs were named GCSPV1 to GCSPV8 (Supplementary Figure S8). Sequence identities of the amplified CP region of the ASPV genome (705 bp excluding primer sequences) between GCSPV1 and GCSPV8 were 75.4–89.7% in terms of nucleotides and 83.8–91.8% in terms of amino acid identities (Table 3). In total, 119 DNA clones were sequenced, and the number of each SV clone was observed to be as follows: 2 (1.7% of the whole) for GCSPV1, 31 (26.1%) for GCSPV2, 10 (8.4%) for GCSPV3, 2 (1.7%) for GCSPV4, 45 (37.8%) for GCSPV5, 12 (10.1%) for GCSPV6, and 14 (11.8%) for GCSPV7, and 3 (2.5%) for GCSPV8. The types and clone numbers of the SVs were different in each infected plant. For example, the type (the number of clones) of GCSPV detected in apple seedlings No. 1–No. 5 were GCSPV2 (13 clones) and GCSPV6 (1 clone) in the No. 1 plant, GCAPV6 (9) and GCAPV7 (9) in the No. 2 plant, GCAPV5 (6), GCAPV6 (1), GCAPV7 (3), and GCAPV8 (3) in the No. 3 plant, GCAPV5 (14) in the No. 4 plant, and GCAPV5 (12), GCAPV6 (1), and GCAPV7 (2) in the No. 5 plant (Table 4). Three SVs (GCSPV1, GCSPV3, and GCSPV4) were detected only from infected *N. occidentalis* plants and not from the infected apple seedlings.

**TABLE 3:** Identities of the nucleotide and amino acid sequences of the coat protein region (705 bp) between the sequence variants (SV) of ASPV found in *N. occidentalis* and apple seedlings inoculated with the RNA transcript of ASPV from AGCD-apple.

	**GCSPV1**	**GCSPV2**	**GCSPV3**	**GCSPV4**	**GCSPV5**	**GCSPV6**	**GCSPV7**	**GCSPV8**
GCSPV1		79.2	89.7	78.2	79.6	83.1	80.9	79.3
GCSPV2	87.2		77.9	82.8	85.8	75.4	78.6	84.1
GCSPV3	91.8	85.4		79.6	80.2	85.5	80.2	80.2
GCSPV4	86.6	89.3	85.5		83.3	76.9	79.6	88.4
GCSPV5	89.7	91.8	88.4	89.3		78.2	80.8	83.5
GCSPV6	88.8	85.5	89.7	84.6	88.0		82.1	78.5
GCSPV7	87.1	86.7	84.1	83.8	89.3	86.7		80.5
GCSPV8	89.3	90.1	87.6	90.6	91.0	86.8	86.7	

**TABLE 4:** Analysis of the sequence variants (GCSPVs) of ASPV found in leaves of apple seedlings (No. 1∼ No. 5) inoculated with ASPV-RNA transcripts and ALSV-AtFT/MdTFL1.

**Plant no. apple seedlings**	**GCSPV (number of clone)**								**No. of clones sequenced**
	**1**	**2**	**3**	**4**	**5**	**6**	**7**	**8**	
1	–	+ (13)	–	–	–	+(1)	−	−	14
2	–	–	–	–	–	+ (9)	+ (4)	–	13
3	–	–	–	–	+ (6)	+ (1)	+ (3)	+ (3)	13
4	–	–	–	–	+ (14)	–	–	–	14
5					+ (12)	+ (1)	+ (2)	–	15
Total no. of clones	0	13	0	0	32	12	9	3	

*The CP region (705 nt) of ASPV genome was amplified by RT-PCR and the DNA products were cloned and sequenced.*

### ASPV-GCSPV2 Probably Induces Green Crinkle Symptoms in the Fruit of Inoculated Apple Seedling

**FIGURE 4:**
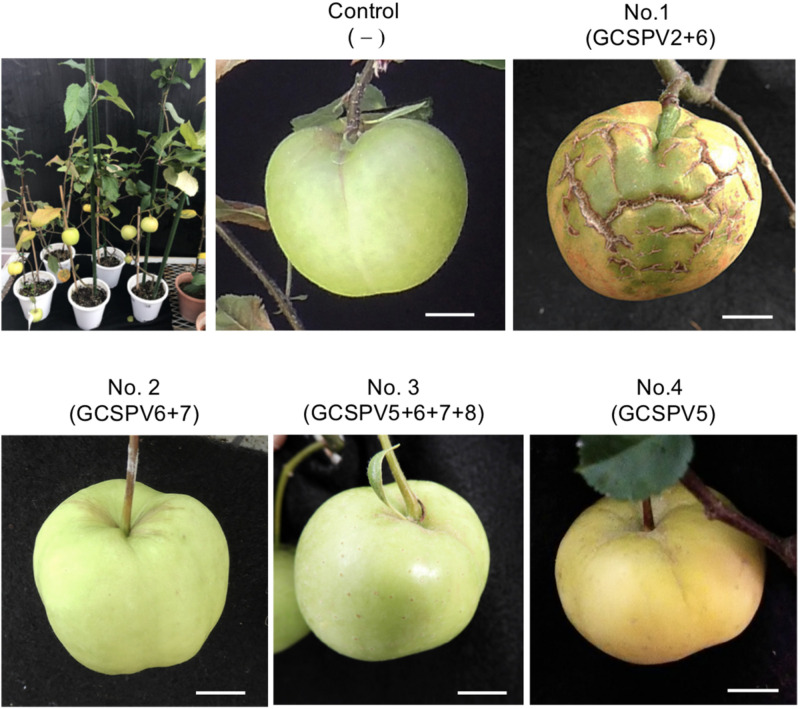
Apple seedlings inoculated with RNA transcripts of ASPV and ALSV-AtFT/MdTFL1 about 10 months after inoculation (upper left) and fruits produced on the inoculated apple seedlings. Control; infected with ALSV-AtFT/MdTFL1 only, No. 1; with GCSPV2 and GCSPV6, No. 2; with GCSPV6 and GCSPV7, No. 3; with GCSPV5, GCSPV6, GCSPV7, and GCSPV8, No. 4 with GCSPV5.

Apple seedlings (No. 1 and No. 5) inoculated with RNA transcripts of ASPV and ALSV-AtFT/MdTFL1 vector flowered 2–3 months after inoculation and produced fruits, as shown in Figure 4. The infected apple seedlings did not show any symptoms on leaves, but a fruit on the No. 1 apple seedling infected with GCSPV2 + GCSPV6 showed cracks on the fruit surface in a manner similar to the symptoms of AGCD (Supplementary Figure S1). Other apple seedlings (Nos. 2, 3, 4, and 5) infected with ASPV-SVs and control seedlings with ALSV-AtFT/MdTFL1 only produced normal shaped apple fruits (Figure 4). We then extracted RNA from the peel and flesh tissues of fruit showing cracks on the apple seedling No. 1, and subjected the RNA to RT-PCR, in which we found that ASPV was distributed in the fruit showing cracks. Cloning and sequence analysis of the amplified DNA products from the fruit indicated that twenty DNA clones from the fruit tissues had the same sequence as GCSPV2, whereas GCSPV6 was not detected in the fruit tissues. Furthermore, apple seedlings Nos. 2, 3, and 5 infected with GCSPV6 showed no symptoms on the fruits (Table 4 and Figure 4). These results strongly suggest that one of the ASPV sequence variants, namely GCSPV2, probably serves as one of the causal agents of AGCD.

**FIGURE 5:**
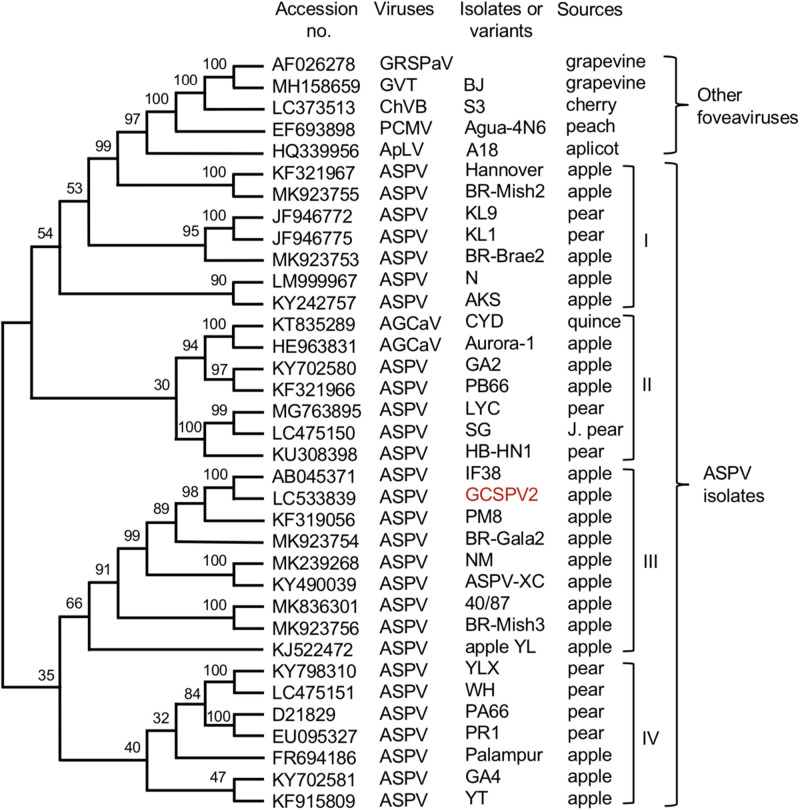
Phylogenetic tree based on the complete nucleotide sequences of ASPV isolates, AGCaV isolates, and five other foveaviruses including grapevine rupestris stem pitting associated virus (GRSPaV), apricot latent virus (ApLV), grapevine virus T (GVT), peach chlorotic mottle virus (PCMV), and cherry virus B (ChVB). A sequence variant analyzed in this study is shown in red. The tree was generated by CLUSTALW program (DNA databank of JAPAN [DDBJ]; URL: http://clustalw.ddbj.nig.ac.jp/). Bootstrap values for each node are shown (1000 replicates).

We have determined the complete nucleotide sequences of the GCSPV2 genomes using DNA clones amplified from a fruit showing symptoms. The genome of GCSPV2 consists of 9294 nt (accession LC533839) excluding the 3′-poly A tail and encodes five proteins from the 5′-terminus; an RNA replication-associated protein (Rep) (248 kDa, 2018 aa), triple gene block (TGB) proteins [TGB1 (25 kDa, 223 aa), TGB2 (13 kDa, 120 aa), TGB3 (8 kDa, 75 aa)] and a CP (42 kDa, 396 aa). Phylogenetic analysis based on the complete nucleotide sequence of 28 isolates of ASPV, 2 of AGCaV, and 5 species of other foveaviruses showed that these ASPV isolates (including AGCaV) could be grouped into 4 clades (I ∼ IV), and that GCSPV2 was most closely related to IF38 from an apple in clade III (Figure 5). The identity of the complete nucleotide sequence between GCSPV2 and IF38 was 93.7% and the identities of the amino acid sequences between GCSPV2 and IF38 were 97.8% (Rep), 97.8% (TGB1), 97.5% (TGB2), 100% (TGB3), and 96.7% (CP). ASPV-IF38 was originally isolated from ‘Fuji’ apple in Iwate University. Morioka, Japan which is not affected with AGCD (Yoshikawa et al., 2001). The nucleotide sequence identities of the genomes between ASPV-GCSPV2 and AGCaV-aurora were 76.6%, and their amino acid identities between them were 88.8% (Rep), 90.6 (TGB1), 84.2% (TGB2), 84.5% (TGB3), and 76.2% (CP).

## Discussion

To investigate viruses in fruit trees, interest in using comprehensive NGS analysis has been increasing in recent years (Coetzee et al., 2010; Ho and Tzanetakis, 2014; Rott et al., 2017; Maliogka et al., 2018). Virome analysis using NGS is expected to be a technique used to search for unknown viral pathogens (Ho and Tzanetakis, 2014; Pecman et al., 2017). For this reason, we first conducted a virome analysis of diseased apple trees in order to determine the types of viruses present in apple trees affected by ARRD and AGCD. Polyacrylamide gel electrophoresis of the dsRNAs extracted from ARRD- and AGCD-infected samples showed several high molecular weight bands as shown in Supplementary Figure S2, which is consistent with the dsRNA patterns of plants infected with ACLSV, ASGV, or ASPV, as reported previously (Yoshikawa and Takahashi, 1988; Magome et al., 1997a; Yoshikawa, 2008). In virome analysis of dsRNAs from diseased samples, we found that five known viruses (ACLSV, ASGV, ASPV, AGCaV, and ApLV) have been infecting both ARRD and AGCD apple trees, and that no new viruses were detected in either type of diseased apples. It was reported that all apple trees with AGCD are infected with ACLSV, ASGV, and/or ASPV (Pacific Northwest Plant Disease Management Handbook^2^; Viral diseases, WUS Tree Fruit^3^). Deep sequencing analysis of an apple sample from Korea showed that these five viruses were identified from eight apple samples showing growth retardation (Cho et al., 2016). In Japan, ACLSV, ASGV, and ASPV have been reported as causative viruses of the top working diseases in apple fruits growing on crab apples (*Malus prunifolia* var. ringo, *M. sieboldii*, and *M. sieboldii arborescens*) as rootstocks (Yanase, 1974). On the other hand, most domesticated orchard apple plants (*M. domestica*) were latently infected with ACLSV, ASGV, and/or ASPV and these are widely distributed as latent viruses in cultivated apples in Japan.

To investigate the association between a specific disease and a particular virus, it is necessary to isolate each virus from the diseased apple trees, and to infect each virus into separate herbaceous plants. In general, leaf extracts of diseased samples were sap-inoculated to different test plants for the separation of target viruses. However, it is difficult to inoculate each apple virus into separate plants because the available hosts (*C quinoa* and *N. occidentalis* etc.) of apple viruses are common for two or three apple viruses (Yanase, 1974; Koganezawa and Yanase, 1990). For that reason, we amplified the full-length DNAs of viral genomic RNAs by RT-PCR using primers containing the T3 promoter sequence, and the full-length viral RNAs were transcribed *in vitro* from the amplified DNAs as templates (Supplementary Figure S3). Inoculation of RNA transcripts into herbaceous plants resulted in a high infection rate (50–100% depending on virus), and we were able to infect each virus coexisting in an ARRD-infected apple tree into separate herbaceous plants (Supplementary Figure S3). This method of using *in vitro* viral RNA transcripts is a very effective method for isolating and separating each virus from fruit trees with multiple viruses, if the entire genome sequences of the target viruses are available. Because the apple russet ring disease is implicated to be caused by a strain (or by strains) of ACLSV (van der Meer, 1986; Desvignes and Boye, 1988; Wood, 2001; Howell et al., 2011), in our subsequent experiments, we considered ACLSV as a candidate for the ARRD causal virus.

For the identification of the pathogenic viruses of diseases, it is important to demonstrate not only the viral species but also their SVs in the diseased trees. The present study indicated that at least seven SVs of ACLSV were found in leaf samples of ARRD-apple (PK-51) (Table 2, Figure 1, and Supplementary Figure S5). Though the data are not shown, multiple SVs were also found for ASGV and ASPV in the ARRD-apple. Similarly, at least eight SVs of ASPV were detected in the leaf samples of AGCD-apple (P-190) (Table 3 and Supplementary Figure S8). Just as multiple virus species gathered in a single tree, each SV in the same virus is likely to have been brought into a single tree by grafting widely used in cultivation of fruit trees in Japan (Magome et al., 1997b). We previously reported the use of identical ALSV labeled with yellow and cyan fluorescence proteins (YFP and GFP) replicated predominantly in discrete areas and separately distributed in leaves coinfected with ALSV-YFP and ALSV-CFP (Takahashi et al., 2007). Spatial separation was also found in plants co-infected with bean yellow mosaic virus (BYMV)-YFP and BYMV-CFP (Takahashi et al., 2007) and plum pox virus (Dietrich and Maiss, 2003), indicating that the separate distribution of virus population may be a universal phenomenon among the same virus species. Considering the distribution of SVs of apple viruses in apple plants, it is assumed that many SVs of each virus are spatially separated from each other in either mosaic or patch form within the tissues and leaves of infected trees. In PK-51 affected by ARRD, there are obvious year-to-year fluctuations in which symptoms appear only for a few fruits in a shoot, or with a relatively large number of symptoms appearing in the same tree. Although temperatures during spring to early summer are reported to have a significant effect on the expression of fruit symptoms (Wood, 1971), another reason for this fluctuation may be due to the interaction between sequence variants coexisted in the infected tree. That is, if the pathogenic variant predominates, symptoms will appear; otherwise, it will not appear at all.

In this study, we reported that one of the SVs of ACLSV (RRACV2), but not other SVs (RRACV1, RRACV3, and RRACV5), caused leaf and fruit symptoms characteristic of ARRD in apple trees. van der Meer (1986) reported that ACLSV isolates from ARRD-apples were graft-transmitted from *C. amaranticolor* to the apple seedlings and some isolates induced russet rings on apple cultivars. He also mentioned that clearly different ACLSV strains can be obtained from the same tree. Desvignes and Boye (1988) also reported that russet rings are very likely to be caused by particular isolates of ACLSV, because back inoculation from *C. quinoa* to ‘Golden Delicious’ was positive. Although these two reports did not characterize ACLSV as a single isolate, the results obtained in this study are consistent with those reports. Wood (2001) reported that at least six distinct strains or forms appear to be present, assuming that all of the russet ring disorders found in New Zealand apple trees are related. Further study is needed to determine whether ACLSV-RRACV2 alone causes ARRD, or if other isolates/strains also cause ARRD. An isolate B6 of ACLSV was isolated from an ARRD-apple different from PK-51 (Yaegashi et al., 2007), and B6 is very closely related to ACLSV-RRACV2 in the phylogenetic tree (Figure 3). It would be interesting to test if B6 induces ARRD in apple trees. As most ACLSV strains or isolates do not cause ARRD in apple (Yanase, 1974; Yaegashi et al., 2011), it is important to identify which genes, or nucleotides, and amino acid sequences in the ACLSV-RRACV2 genome are involved in the induction of leaf and fruit symptoms characteristic to ARRD.

For AGCD, it is assumed that ASPV or AGCaV may be a causal virus (Howell et al., 2011; James et al., 2013); however, there are no reports of back-inoculation of the isolated virus to apple plants. In AGCD, the symptoms appear only on the fruits and not on the leaves, so we must observe the fruits of the back-inoculated apple plants. In this study, we used VIF technology using the ALSV vector to accelerate apple fruit formation, because it takes at least 6–7 years for apple seedlings to blossom. ALSV does not induce any symptoms on leaves and fruits (Yamagishi et al., 2014).

The apple tree (P-190) affected by AGCD tested in this study showed symptoms in almost all fruits every year. Our results indicated that the apple tree (P-190) with AGCD has a complex viral infection because the plant contained at least eight SVs (GCSPV1- GCSPV8) (Table 3), even for ASPV alone. The type and proportion of SVs were different among the inoculated plants, even when using the same RNA transcript as inoculum (Table 4). We showed that one of the sequence variants (GCSPV2) probably induces severe cracks on the fruit surface (apple seedling No. 1 in Figure 4), which is very similar to the AGCD symptoms shown in Supplementary Figure S1. Though the apple seedling No. 1 was infected with both GCSPV2 and GCSPV6 (Table 4), most of the variants detected were GCSPV2 (13/14 clones) (Table 4) and only GCSPV2 was detected from fruit showing cracks (20/20 clones). Furthermore, apple plants infected with other SVs including GCSPV6 produced normal-shaped apple fruits (Table 4 and Figure 4). ASPV-GCSPV2 was distributed in the fruit and showed cracks, strongly suggesting that ASPV-GCSPV2 might be one of the AGCD causal agents. We plan to inoculate the virus-free ‘Golden Delicious’ trees with branches of infected trees (No. 1 to No. 5) by grafting in ARRD to further confirm the ASPV-SVs pathogenicity this year.

Since Jelkmann (1994) first reported the nucleotide sequence of the pear ASPV genome, the complete nucleotide sequences of many isolates from apple, pear, and quince have been reported, as shown in Figure 5. There is a great genetic diversity among ASPV isolates, and some isolates had less than about 72% nt identity (or 80% aa identity) between their CP or polymerase genes, which corresponds to the species demarcation criteria in the *Foveavirus* genus (Youssef et al., 2011; Adams et al., 2012). James et al. (2013) reported that a putative new foveavirus (AGCaV), or a variant or strain of ASPV was isolated from an Aurora Golden Gala apple showing severe symptoms of AGCD. A phylogenetic analysis based on the complete nucleotide sequences showed that ASPV isolates might be grouped into 4 clades, and that GCSPV2 was in clade III with IF38 from apple (Figure 3). On the other hand, AGCaV (aurora-1) was grouped into clade II (Figure 3), suggesting that multiple ASPV variants including AGCaV may cause AGCD. It will be interesting to determine which gene(s) in the ASPV genome are involved in the induction of fruit symptoms of AGCD.

As previously mentioned, there are virus-like diseases of deciduous fruit trees whose causal agents are still not identified. The strategy for fulfillment of the Koch’s Postulates presented here (Supplementary Figure S9) can provide a system to prove if the virus found in diseased tissues is the same as the pathogen causing diseases in fruit trees.

## Data Availability Statement

The datasets presented in this study can be found in online repositories. The names of the repository/repositories and accession number(s) can be found in the article/Supplementary Material.

## Author Contributions

CL amplified the full-length DNA, transcribed infectious RNAs, and analyzed the sequence variants of ASPV. HY conducted a phylogenetic analysis of ACLSV and ASPV. RK amplified the full-length DNA, transcribed infectious RNAs, and analyzed the sequence variants of ACLSV. AK analyzed the viruses of apple plants back-inoculated with ACLSV and ASPV and determined the complete nucleotide sequence of the ASPV-GCSPV2 genome. NYa carried out the inoculation of virus RNA to apple plants by particle bombardment and determined the complete nucleotide sequences of the ACLSV-RRACV1 and RRACV2 genome. TI conducted the graft-transmission tests. NYo is the principal investigator, and he supervised the experiments and wrote the manuscript. All the authors contributed to the article and approved the submitted version.

## Conflict of Interest

The authors declare that the research was conducted in the absence of any commercial or financial relationships that could be construed as a potential conflict of interest.

## Footnotes

1 https://blast.ncbi.nlm.nih.gov/Blast.cgi

2 https://pnwhandbooks.org/node/2133

3 http://treefruit.wsu.edu/web-article/viral-diseases/
