# Supplementary material for: Apple Russet Ring and Apple Green Crinkle Diseases: Fulfillment of Koch’s Postulates by Virome Analysis, Amplification of Full-Length cDNA of Viral Genomes, in vitro Transcription of Infectious Viral RNAs, and Reproduction of Symptoms on Fruits of Apple Trees Inoculated With Viral RNAs
Source: Front Microbiol. 2020 Jul 10;11:1627. doi: 10.3389/fmicb.2020.01627 (PMC7365870; doi:10.3389/fmicb.2020.01627)
Supplement: Supplementary file 1 [file Presentation_1.PPTX]

## Slide 1
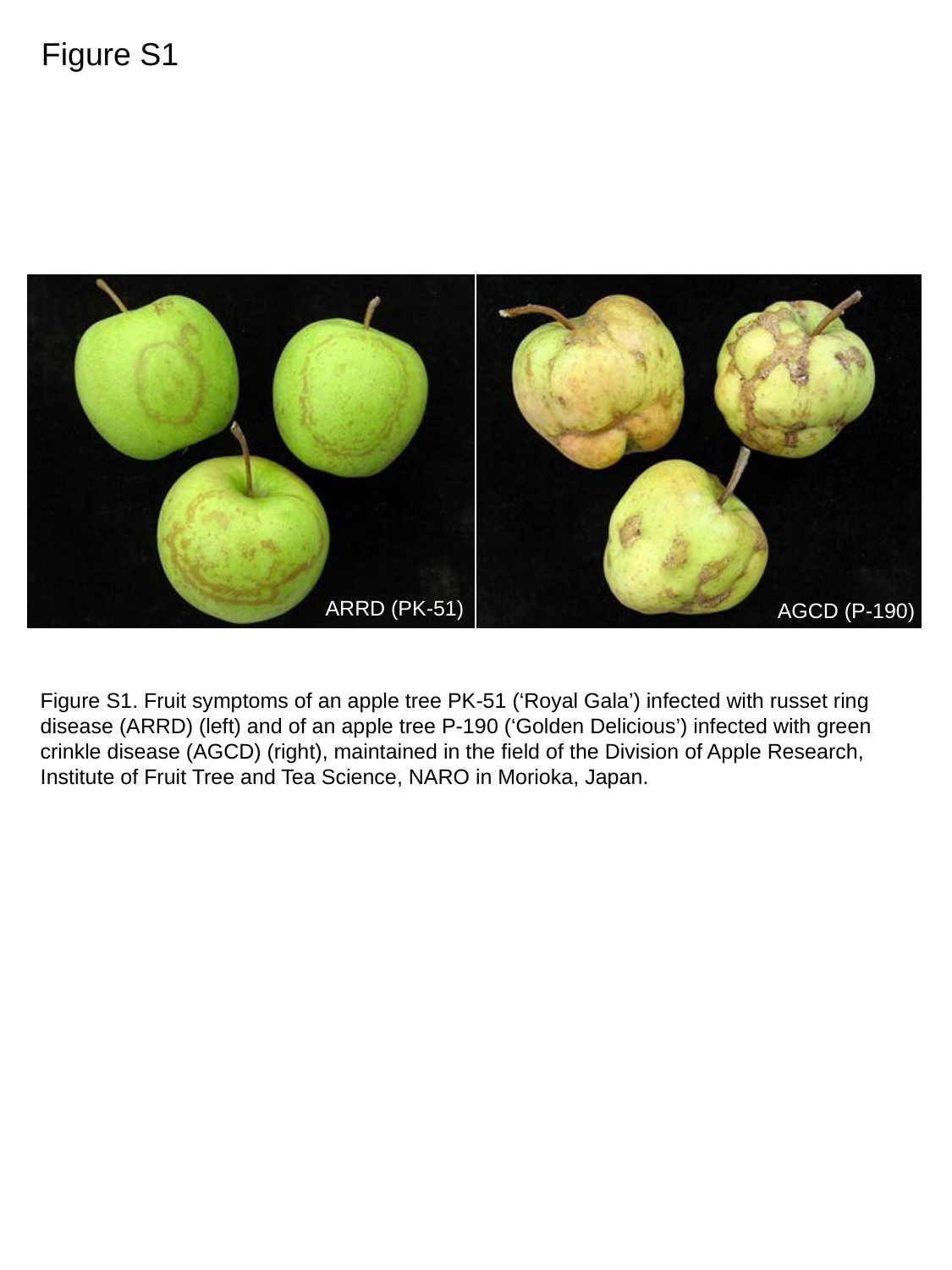

Figure S1
ARRD (PK-51)
AGCD (P-190)
Figure S1. Fruit symptoms of an apple tree PK-51 (‘Royal Gala’) infected with russet ring disease (ARRD) (left) and of an apple tree P-190 (‘Golden Delicious’) infected with green crinkle disease (AGCD) (right), maintained in the field of the Division of Apple Research, Institute of Fruit Tree and Tea Science, NARO in Morioka, Japan.

## Slide 2
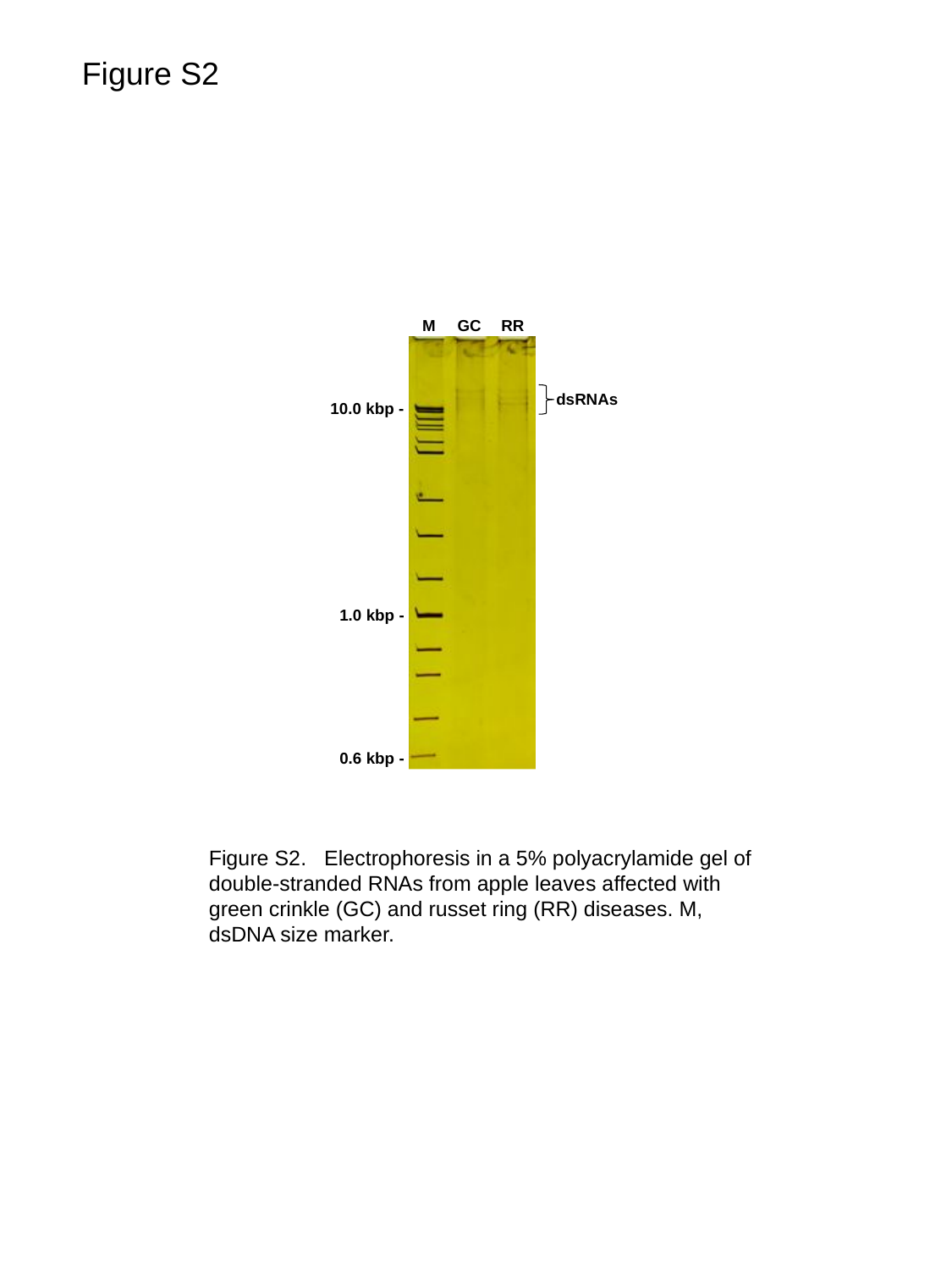

Figure S2
M
GC
RR
dsRNAs
10.0 kbp -
1.0 kbp -
0.6 kbp -
Figure S2. Electrophoresis in a 5% polyacrylamide gel of double-stranded RNAs from apple leaves affected with green crinkle (GC) and russet ring (RR) diseases. M, dsDNA size marker.

## Slide 3
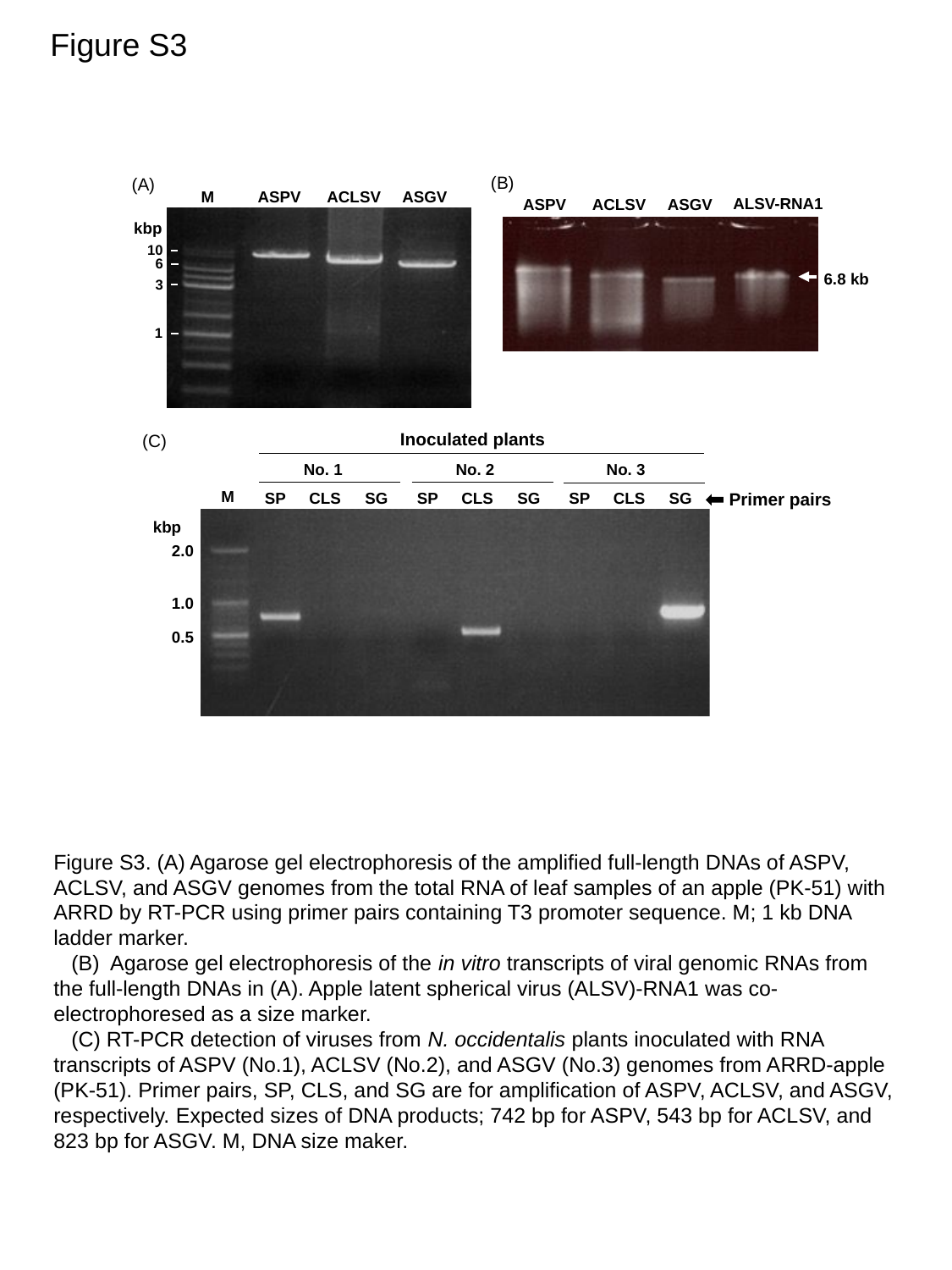

Figure S3
(B)
ALSV-RNA1
　 ASPV ACLSV ASGV
6.8 kb
(A)
 M ASPV ACLSV ASGV
kbp
10
6
3
1
Inoculated plants
No. 1
No. 2
No. 3
M
SP
CLS
SG
kbp
2.0
1.0
0.5
SP
CLS
SG
Primer pairs
SP
CLS
SG
(C)
Figure S3. (A) Agarose gel electrophoresis of the amplified full-length DNAs of ASPV, ACLSV, and ASGV genomes from the total RNA of leaf samples of an apple (PK-51) with ARRD by RT-PCR using primer pairs containing T3 promoter sequence. M; 1 kb DNA ladder marker.
 (B) Agarose gel electrophoresis of the in vitro transcripts of viral genomic RNAs from the full-length DNAs in (A). Apple latent spherical virus (ALSV)-RNA1 was co-electrophoresed as a size marker.
 (C) RT-PCR detection of viruses from N. occidentalis plants inoculated with RNA transcripts of ASPV (No.1), ACLSV (No.2), and ASGV (No.3) genomes from ARRD-apple (PK-51). Primer pairs, SP, CLS, and SG are for amplification of ASPV, ACLSV, and ASGV, respectively. Expected sizes of DNA products; 742 bp for ASPV, 543 bp for ACLSV, and 823 bp for ASGV. M, DNA size maker.

## Slide 4
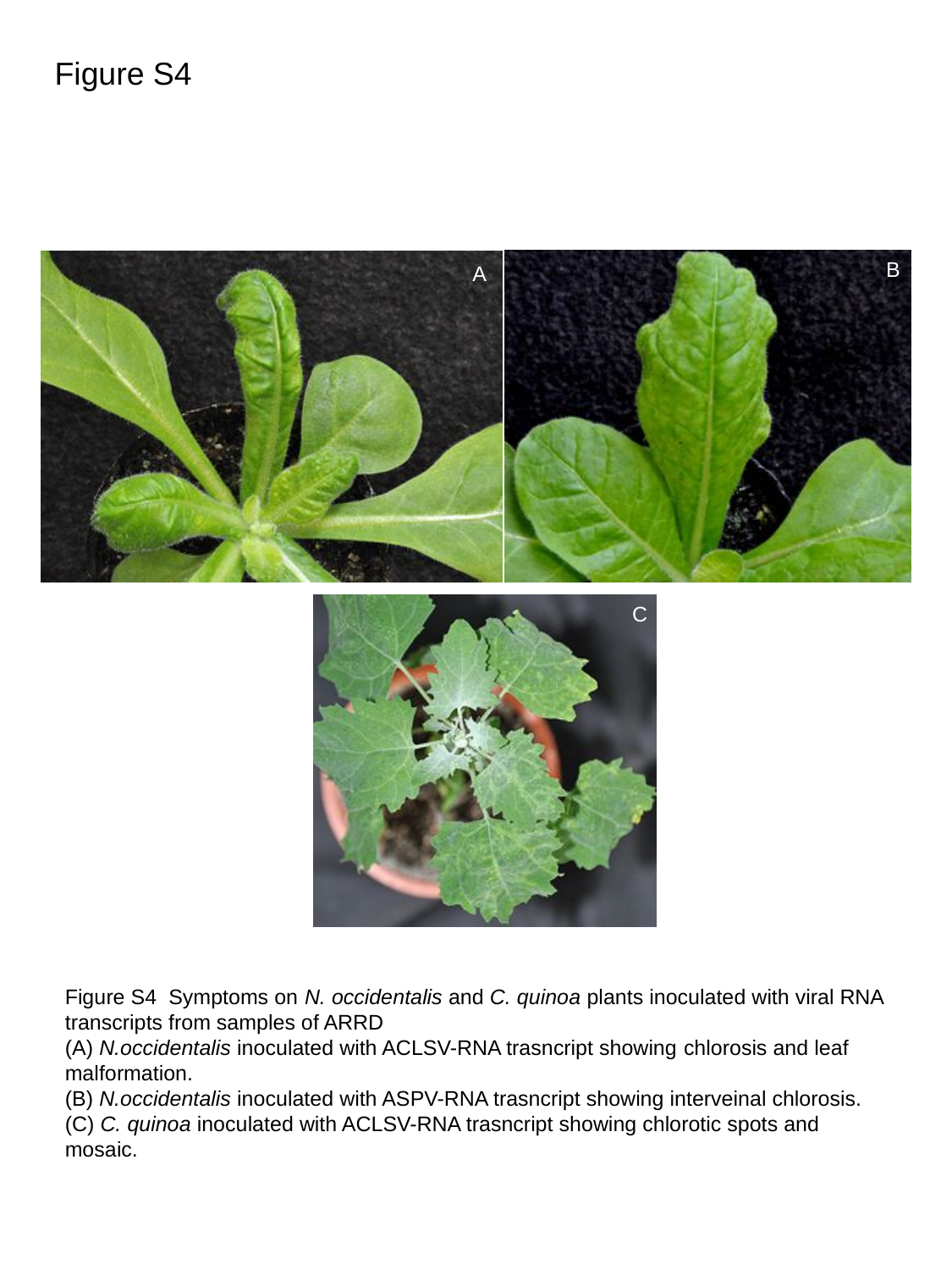

Figure S4
B
A
C
Figure S4 Symptoms on N. occidentalis and C. quinoa plants inoculated with viral RNA transcripts from samples of ARRD
(A) N.occidentalis inoculated with ACLSV-RNA trasncript showing chlorosis and leaf malformation.
(B) N.occidentalis inoculated with ASPV-RNA trasncript showing interveinal chlorosis.
(C) C. quinoa inoculated with ACLSV-RNA trasncript showing chlorotic spots and mosaic.

## Slide 5
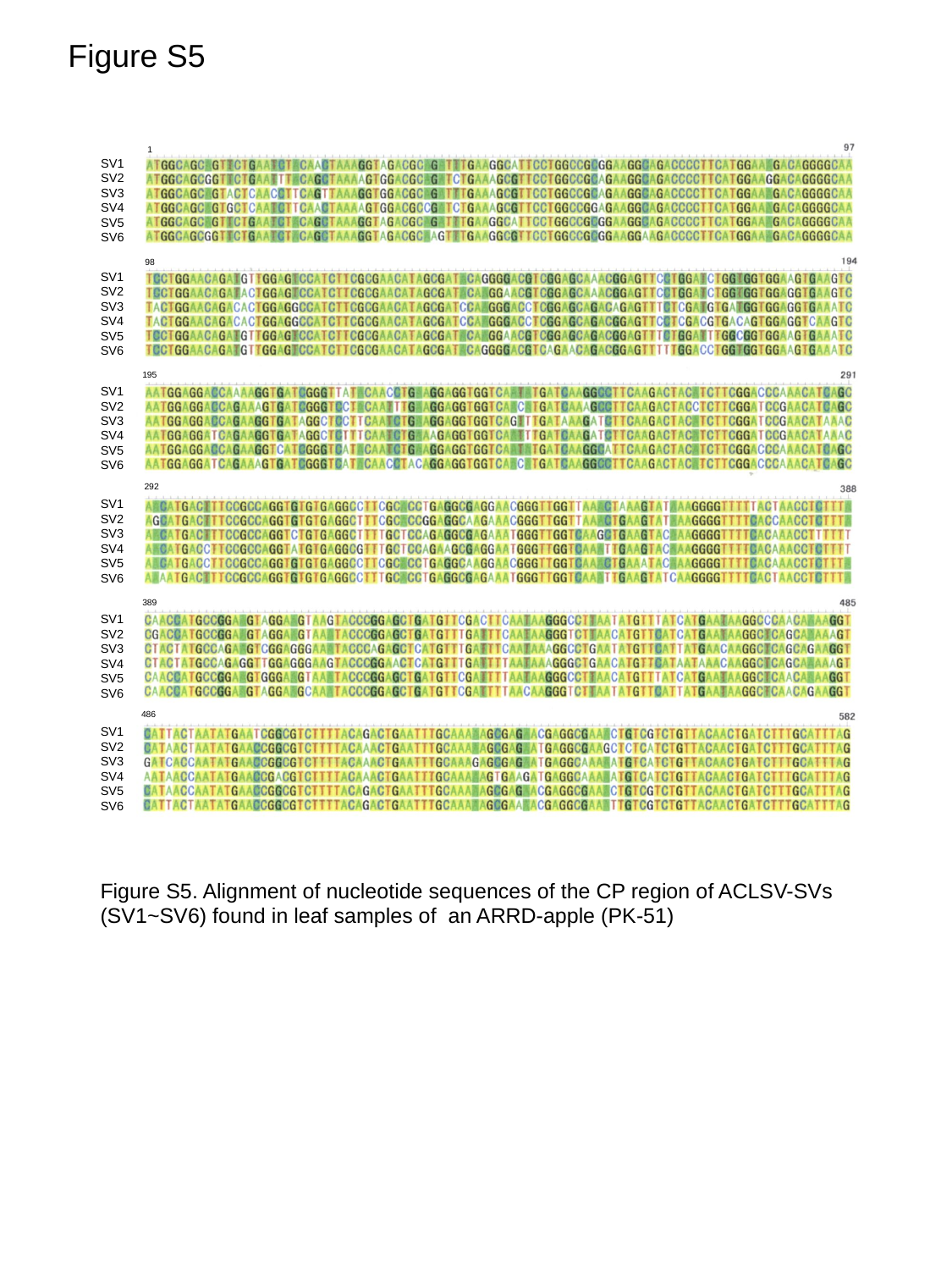

Figure S5
1
SV1
SV2
SV3
SV4
SV5
SV6
98
SV1
SV2
SV3
SV4
SV5
SV6
195
SV1
SV2
SV3
SV4
SV5
SV6
292
SV1
SV2
SV3
SV4
SV5
SV6
389
SV1
SV2
SV3
SV4
SV5
SV6
486
SV1
SV2
SV3
SV4
SV5
SV6
Figure S5. Alignment of nucleotide sequences of the CP region of ACLSV-SVs (SV1~SV6) found in leaf samples of an ARRD-apple (PK-51)

## Slide 6
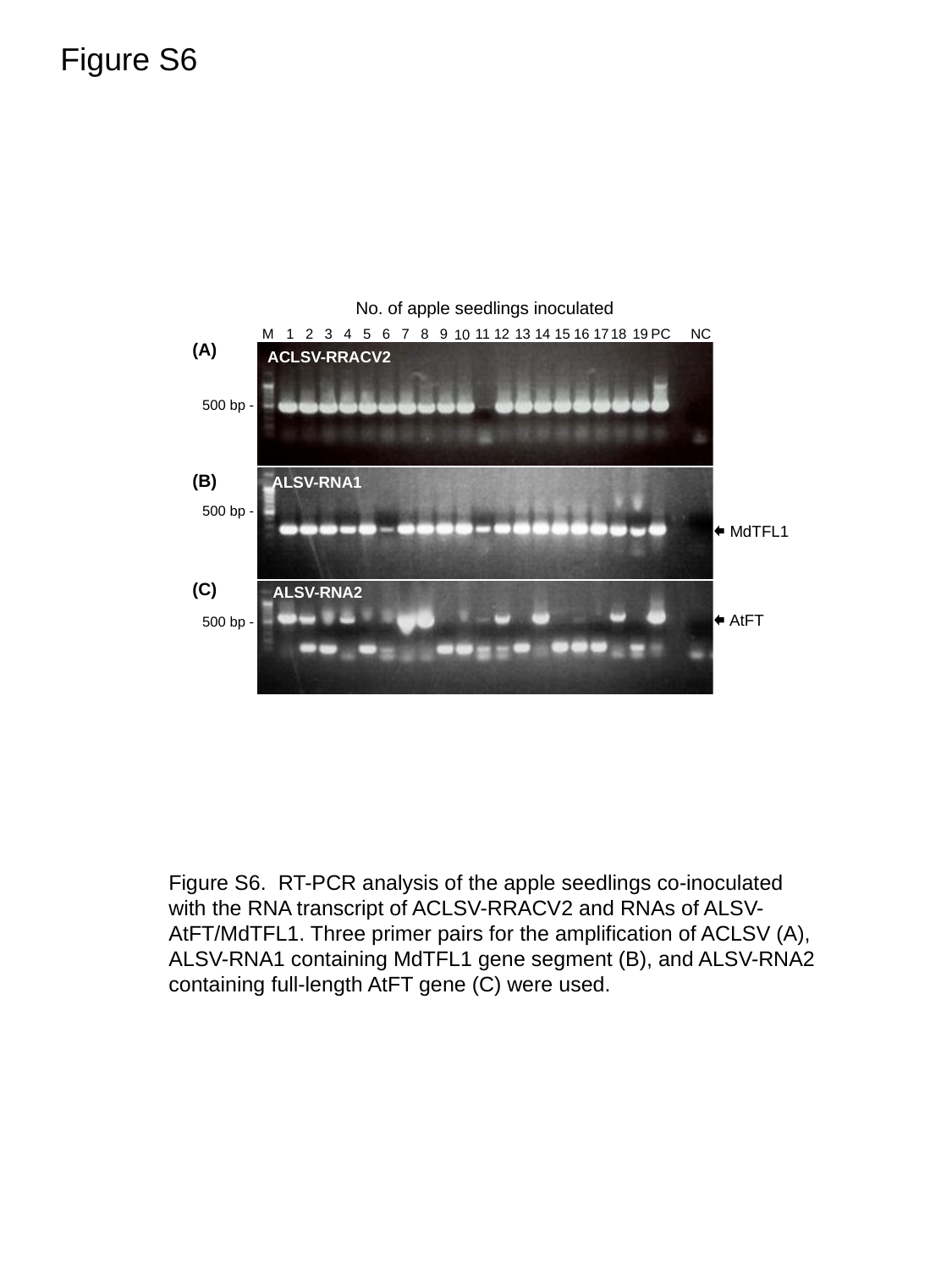

Figure S6
No. of apple seedlings inoculated
M
1
2
3
4
5
6
7
8
9
11
12
13
14
15
16
17
18
19
PC
NC
10
ACLSV-RRACV2
500 bp -
ALSV-RNA1
500 bp -
MdTFL1
ALSV-RNA2
AtFT
500 bp -
(A)
(B)
(C)
Figure S6. RT-PCR analysis of the apple seedlings co-inoculated with the RNA transcript of ACLSV-RRACV2 and RNAs of ALSV-AtFT/MdTFL1. Three primer pairs for the amplification of ACLSV (A), ALSV-RNA1 containing MdTFL1 gene segment (B), and ALSV-RNA2 containing full-length AtFT gene (C) were used.

## Slide 7
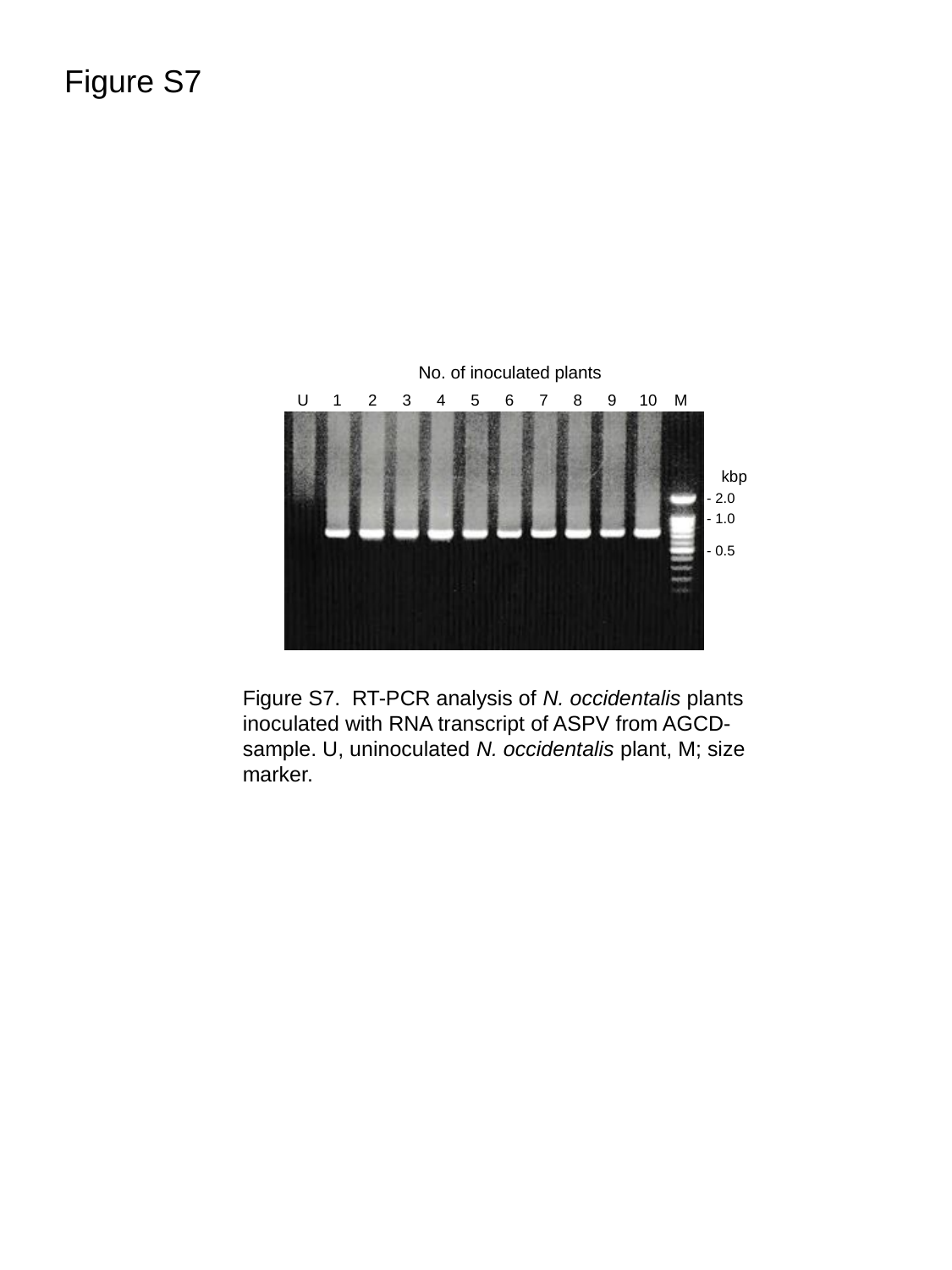

Figure S7
No. of inoculated plants
U
1
2
3
4
5
6
7
8
9
10
M
kbp
- 2.0
- 1.0
- 0.5
Figure S7. RT-PCR analysis of N. occidentalis plants inoculated with RNA transcript of ASPV from AGCD-sample. U, uninoculated N. occidentalis plant, M; size marker.

## Slide 8
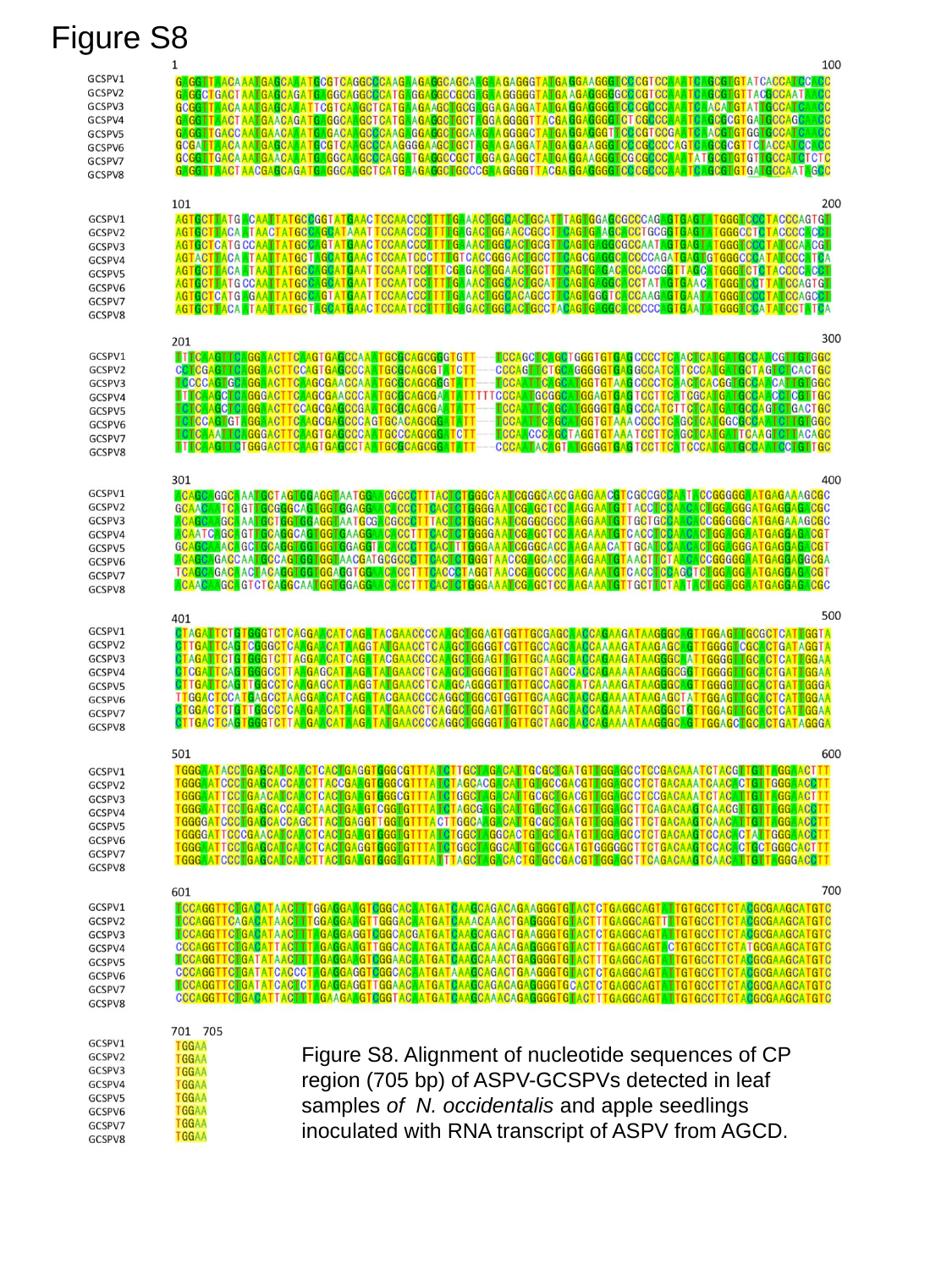

Figure S8
Figure S8. Alignment of nucleotide sequences of CP region (705 bp) of ASPV-GCSPVs detected in leaf samples of N. occidentalis and apple seedlings inoculated with RNA transcript of ASPV from AGCD.

## Slide 9
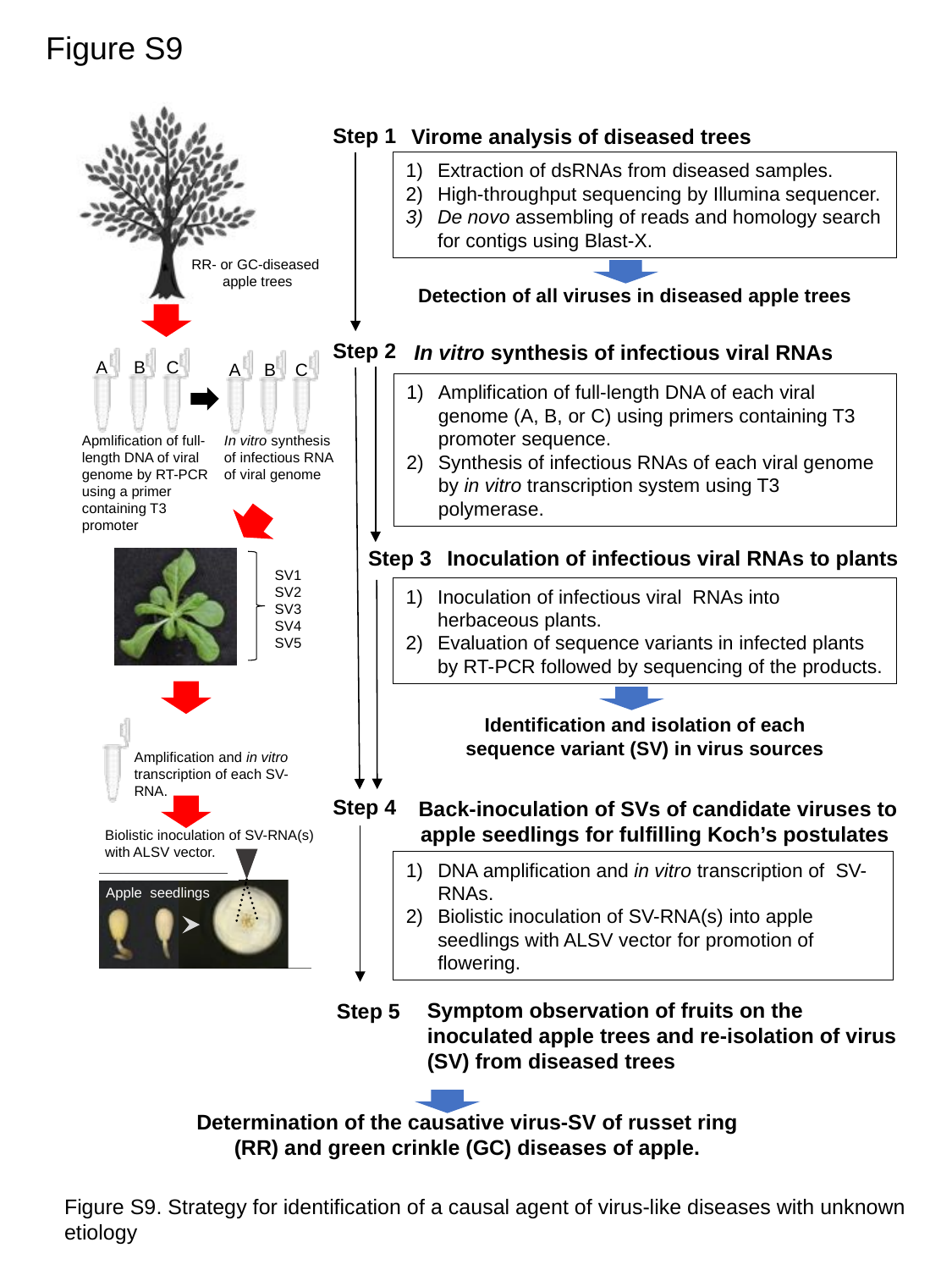

Figure S9
RR- or GC-diseased
apple trees
Step 1
Virome analysis of diseased trees
Extraction of dsRNAs from diseased samples.
High-throughput sequencing by Illumina sequencer.
De novo assembling of reads and homology search for contigs using Blast-X.
Detection of all viruses in diseased apple trees
Step 2
In vitro synthesis of infectious viral RNAs
A
B
C
A
B
C
In vitro synthesis of infectious RNA of viral genome
Apmlification of full-length DNA of viral genome by RT-PCR using a primer containing T3 promoter
Amplification of full-length DNA of each viral genome (A, B, or C) using primers containing T3 promoter sequence.
Synthesis of infectious RNAs of each viral genome by in vitro transcription system using T3 polymerase.
Step 3
Inoculation of infectious viral RNAs to plants
SV1
SV2
SV3
SV4
SV5
Inoculation of infectious viral RNAs into herbaceous plants.
Evaluation of sequence variants in infected plants by RT-PCR followed by sequencing of the products.
Identification and isolation of each sequence variant (SV) in virus sources
Amplification and in vitro transcription of each SV-RNA.
Step 4
Back-inoculation of SVs of candidate viruses to apple seedlings for fulfilling Koch’s postulates
Biolistic inoculation of SV-RNA(s) with ALSV vector.
Apple seedlings
DNA amplification and in vitro transcription of SV-RNAs.
Biolistic inoculation of SV-RNA(s) into apple seedlings with ALSV vector for promotion of flowering.
Symptom observation of fruits on the inoculated apple trees and re-isolation of virus (SV) from diseased trees
Step 5
Determination of the causative virus-SV of russet ring (RR) and green crinkle (GC) diseases of apple.
Figure S9. Strategy for identification of a causal agent of virus-like diseases with unknown etiology
