# Supplementary material for: Apple Russet Ring and Apple Green Crinkle Diseases: Fulfillment of Koch’s Postulates by Virome Analysis, Amplification of Full-Length cDNA of Viral Genomes, in vitro Transcription of Infectious Viral RNAs, and Reproduction of Symptoms on Fruits of Apple Trees Inoculated With Viral RNAs
Source: Front Microbiol. 2020 Jul 10;11:1627. doi: 10.3389/fmicb.2020.01627 (PMC7365870; doi:10.3389/fmicb.2020.01627)
Supplement: Supplementary file 2 [file Table_1.DOCX]

Table S1. Primers used for the determination of the nucleotide sequences of ACLSV-RRACV1 and ACLSV-RRACV2

| Primer pairs | Sequence (5’ – 3’) | Nucleotide positions of the genome amplified |
| --- | --- | --- |
| ACLSV 5end | TGATACTGATACAGTGTACACT | 1 - 773 |
| ACLSV 755-773 R | CATGAATCTCAAACTGGTA |  |
| ACLSV 689-707 F | TGGGCCACTCTGGTTTTTC | 688 - 1493 |
| ACLSV 1473-1493 R | GTTCAAGCCATTCCTTCCTTC |  |
| ACLSV 1400-1419 F | AGGATGGTCATAGATCAGAT | 1400 - 2331 |
| ACLSV 2309-2331 R | GTTGGGTAGGAAACTTTGTCATG |  |
| ACLSV 2198-2217 F | GTTGGGAATGATTACCTTGC | 2198 - 3030 |
| ACLSV 3009-3030 R | ACATGATCCAAGACTCCATTTG |  |
| ACLSV 2811-2831 F | GTTCACTTGATGACGCAAAAG | 2811 - 3748 |
| ACLSV 3728-3748 R | GTTGATCCTGTAGCTGTACCA |  |
| ACLSV 3647-3668 F | TACTTCTGTCCTAAGGATGACA | 3647 - 4440 |
| ACLSV 4421-4440 R | CCTTTGAATTCCCTGAATTC |  |
| ACLSV 4229-4248 F | GATGAAATGGACAGAGAAGC | 4229 - 5184 |
| ACLSV 5162-5184 R | CATTTCATTTTGATGTAACTCTG |  |
| ACLSV 5093-5112 F | CAGGATCATACCATTTTGGC | 5093 - 6127 |
| ACLSV 6109-6127 R | GAACCACTGTCCAGATTGA |  |
| ACLSV 5830-5851 F | CTTCTGACCTGATGATTCATTG | 5830 - 6401 |
| ACLSV 6385-6401 R | CTCACTTGATCGTCCTGTTC |  |
| ACLSV 6069-6086 F | TGGGAAACATTTGAGCAG | 6069 - 6805 |
| ACLSV 6787-6805 CP R | GATTCAGAACTGCTGCCAT |  |
| AC-CP 6732-6755 F | AACATTTCTGCAAGAGAGTTTCAG | 6732 - 7553 |
| ACLSV 3’end t6 | TTTTTTGTAGTAAAATATTTAAAAGT |  |

Primer sequences were designed based on the nucleotide sequence of ACLSV-B6 genome.

Table S2. Primers used for the determination of the nucleotide sequences of ASPV-GCSPV2

| Primer pairs | Sequence (5’ – 3’) | Nucleotide positions of the genome amplified |
| --- | --- | --- |
| SP1+ | GGATACGCAAACAAACTCTG | 1 - 528 |
| SP528- | AAAGCCTGTTCTTCTGTCAC |  |
| SP399+ | GATCTTCAAATGGTGGAAAC | 399 - 1394 |
| SP1394- | TTAATGAATTCTAGATCGTC |  |
| SP1201+ | AACATGCTTCCTGACATGCG | 1201 - 2310 |
| SP2310- | GTCCAGGAAATCTGGCCAAC |  |
| SP2200+ | TTTATAGGAGGTGCTCGTGG | 2200 - 3224 |
| SP3224- | ATTTTTGGCTTCAGAAAGGC |  |
| SP3084+ | TGGTGCAATGCATGTAGTTG | 3084 - 4068 |
| SP4068- | ATCCAAAAGAGTGGAAGCAC |  |
| SP3925+ | ATCCCTGTCCTGTCCAGAAG | 3925 - 4968 |
| SP4968- | TATGAAGTGAATGTCATGGC |  |
| SP4800+ | AATTGTGGCAGCACATCTTG | 4800 - 5792 |
| SP5792- | AGAGTTTGTCCAGCCTTAGC |  |
| SP5653+ | AAAGCATGGCCACAATAGAG | 5654 - 6700 |
| SP6698- | ATATGCTTACACATGAATTC |  |
| SP6571+ | AACATCCGTGATCTCTTCAG | 6571 - 7590 |
| SP7588- | CTCTTTTGGTCCACAGTAGC |  |
| SP7479+ | CTCTGACCAGAAGCACACTG | 7481 - 8628 |
| SP8626- | TTGCTGGCAACGACCCCAGC |  |
| SP8148+ | TTCGACCCTAACCTTCATGG | 8150 - 8892 |
| SP8890- | CTTTGAGTTTGCAGCATGTT |  |

Primer sequences were designed based on the nucleotide sequence of ASPV-IF38 genome.

Table S3. Comparison of the nucleotide sequence between DNAs amplified from the grafted apple trees and RRACVs used for inoculation.

| Apple trees from which DNA were amplified | Inoculated RRACVs | | | | |
| --- | --- | --- | --- | --- | --- |
|  | RRACV1 | RRACV2 | RRACV3 | RRACV4 | RRACV5 |
| APT1 | 99.1 | 91.2 | 85.1 | 83.7 | 85.2 |
| APT2 | 91.3 | 99.7 | 86.9 | 85.6 | 85.3 |
| APT3 | 85.0 | 87.0 | 99.7 | 91.7 | 92.0 |
| APT1+4 | 99.4 | 91.3 | 85.3 | 83.8 | 85.0 |
| APT5 | 85.0 | 85.6 | 92.3 | 91.2 | 99.6 |

* The CP region (582 bp) of six cDNA clones from each apple tree inoculated with RRACVs was sequenced. The nucleotide sequence identities among six cDNA clones from each apple were more than 99% in all SVs.
